# Genetic predisposition to serum 25 hydroxyvitamin D concentrations does not influence the risk of decreasing celiac disease in European ancestry: Evidence from meta-analysis and Mendelian randomization

**DOI:** 10.1097/MD.0000000000049587

**Published:** 2026-07-03

**Authors:** Mahdi Akbarzadeh, Fahimeh Haghighatdoost, Amirhossein Ataei Kachouei, Alireza Soleymani Taloubaghi, Taha Rafiei, Soheil Rahmati, Yasamin Jiani, Amir Hesam Saeidian, Fereidoun Azizi, Mehdi Hedayati, Maryam S. Daneshpour, Mohammad Rafiei, Danial Habibi

**Affiliations:** a Cellular and Molecular Endocrine Research Center, Research Institute for Endocrine Molecular Biology, Research Institute for Endocrine Sciences, Shahid Beheshti University of Medical Sciences, Tehran, Iran; b Isfahan Cardiovascular Research Center, Cardiovascular Research Institute, Isfahan University of Medical Sciences, Isfahan, Iran; c Faculty of Life Sciences: Food, Nutrition and Health, University of Bayreuth, Kulmbach, Germany; d Data Science and AI Applications, Graduate School of Engineering Science and Technology, National Yunlin University of Science and Technology, Douliu, Taiwan; e School of Medicine, Arak University of Medical Sciences, Arak, Iran; f School of Medicine, Mazandaran University of Medical Sciences, Ramsar, Iran; g Houston Healthcare Medical Center, Houston, TX; h Department of Surgery, Rasool-E Akram Hospital School of Medicine, Iran University of Medical Sciences, Tehran, Iran; i Endocrine Research Center, Research Institute for Endocrine Sciences, Shahid Beheshti University of Medical Sciences, Tehran, Iran; j Department of Biostatistics, school of Medicine, Arak University of Medical sciences, Arak, Iran; k Cellular and Molecular Biology Research Center, Health Research Institute, Babol University of Medical Sciences, Babol, Iran.

**Keywords:** Bayesian Meta-analysis, celiac disease, GSMR, meta-analysis, serum 25 hydroxyvitamin D concentration, two-sample Mendelian randomization

## Abstract

While prior research has indicated a possible association between serum 25 hydroxyvitamin D [25(OH)D] and celiac disease (CD), key questions remain unresolved without clear consensus. To assess association, we perform Mendelian randomization and a systematic review and meta-analysis of existing studies in European ancestry. A systematic review and meta-analysis performed by using both standardized mean difference and a Bayesian framework. Moreover, Mendelian randomization conducted via Generalized Summary-data-based Mendelian randomization (GSMR) and two-sample Mendelian randomization. Data on serum 25(OH)D levels were obtained from the Medical Research Council’s Integrative Epidemiology Unit (GWAS ID: ebi-a-GCST90000618; N = 4,96,946), while data on CD included 11,812 cases and 229 controls (GWAS ID: ebi-a-GCST005523). The main method used was the random inverse variance weighted (IVW) approach, supplemented by various sensitivity analyses to strengthen the findings. Both the meta-analysis and Mendelian randomization analysis indicate that vitamin D levels have no effect on the risk of developing celiac disease. Using a random-effects model, the standardized mean difference was calculated as −0.79 (95% CI: −2.02, 0.45), which was not statistically significant (*T*_Statistic_=−1.77, *P* = .152). The Bayesian meta-analysis reinforced these results, showing a point estimate of −0.6 with a wide 95% credible interval of (−13.6, 13.2), highlighting substantial uncertainty regarding the true effect size. The GSMR also found no significant association between 25(OH)D levels and CD (OR = 0.988; 95% CI: 0.853–1.145; *P* = .878). This null finding was corroborated by two-sample Mendelian randomization (OR_IVW_ = 0.72, 95% CI: 0.49–1.05, *P* = .091). The Steiger test confirmed the absence of a directional relationship, and cluster analysis revealed no heterogeneity patterns (OR_IVW_ = 0.374, 95% CI: 0.238–0.588). These findings were consistent across multiple MR methods, including penalized MR, robust MR, penalized robust MR, MR-Egger (both t-distribution and radial approaches), SIMEX, MR-PRESSO, Maximum likelihood, MR-Mix, RAPS, MR-Least Absolute Shrinkage and Selection Operator, debiased IVW, and contamination mixture models. By questioning the presumed direct connection between 25(OH)D and CD, it appears that 25(OH)D may not be a primary contributor to CD, which could impact future prevention approaches and alter clinical guidelines.

## 1. Introduction

Vitamin D (VitD) performs diverse functions that extend beyond its role as just a vitamin, such as the modulation of calcium absorption and the regulation of immune system responses.^[1,2]^ The vitamin D receptor (VDR), classified as a nuclear hormone receptor, orchestrates the biological functions of 1,25(OH)2D3, the active form of VitD. The VDR has been demonstrated to be present on antigen-presenting cells and lymphocytes, underscoring VitD’s potential function as a key regulator of immune and inflammatory responses.^[3,4]^ Research indicates that VitD may exhibit a shielding role in maintaining the integrity of the intestinal barrier by influencing the expression of tight junction proteins, amending the composition of gut microbiota, and controlling the synthesis of antimicrobial peptides. This underlines the importance of VitD in both the onset and treatment of gastrointestinal immune disorders.^[4,5]^

Celiac disease (CD) is a chronic autoimmune condition that influences multiple organs and is provoked by the consumption of gluten in genetically prone individuals who possess HLA-DQ2/8 alleles.^[4,6]^ CD is estimated to have a global prevalence of approximately 0.7%, with marked increases noted in particular in developed countries.^[2,7]^ Subjects with CD experience changes in the intestinal mucosa due to the disruption of tight junctions between enterocytes. Such disruption can cause malnutrition, diarrhea, and a spectrum of nutritional deficiencies.^[8]^ The main approach to treating CD is strict compliance with a lifelong gluten-free diet (GFD).^[9]^

Approximately 25 years ago, Corazza et al discovered that patients with CD had lower VitD levels compared to healthy subjects.^[10]^ Since that time, several studies have explored the association between VitD and CD, which yielded inconsistent findings. Research has indicated that CD is more common among individuals living in northern areas, where there is reduced exposure to sunlight.^[11]^ In genetically susceptible individuals, a deficiency in VitD could play a substantial role in the initiation of CD in children. In particular, VitD deficiency can disrupt immunological balance, possibly leading to alterations in the intestinal mucosa and heightened susceptibility to acute gastrointestinal infections.^[12]^ In contrast, research by Villanueva et al revealed no notable difference in VitD levels between children diagnosed with CD and those who were not affected.^[13]^ A meta-analysis that included 1137 patients with CD and 2613 controls demonstrated significantly reduced VitD levels in individuals with CD. After implementing GFD, the VitD levels in treated patients increased, approaching those of healthy subjects.^[14]^ The indication for routine VitD supplementation in CD is also controversial across different guidelines.^[15]^ Ciacci et al discovered that concentrations of 25(OH)D3 and 1,25(OH)2D3 in subjects with CD, despite being below the normal range, did not correlate with reduced bone mineral density, which raises doubts about the indication for VitD supplementation for all adults diagnosed with CD.^[16]^ Moreover, a cohort study carried out in Norway investigated the impact of VitD supplementation in the first 18 months, as well as prenatal supplementation, on the likelihood of developing CD, revealing no significant association.^[17]^ Considering the conflicting findings produced by the previous studies, no decision has been reached regarding the efficacy of VitD supplementation in CD subjects.

Although previous research suggested a potential connection between VitD levels and the initiation and progression of CD, numerous facets of this relationship remain unclear, and no agreement has been reached. Mendelian randomization (MR) is a cutting-edge analytical technique that utilizes data from genome-wide association studies (GWAS) to employ genetic variants, aiming to shed light on the causal relationships between different exposures and outcomes. MR employs genetic variations as proxies for prolonged exposures, and since these genetic variations are preestablished, this method is less susceptible to biases arising from confounding factors, reverse causation, and measurement inaccuracies. In this research, we employed SNPs identified through GWAS as instrumental variables to perform 2-sample MR analyses, aiming to explore the possible causal link between VitD and CD. In parallel, a systematic review and meta-analysis of observational studies was conducted to quantitatively compare 25(OH)D3 concentrations between individuals diagnosed with CD and non-affected controls.

## 2. Materials and method

**Figure 1.**
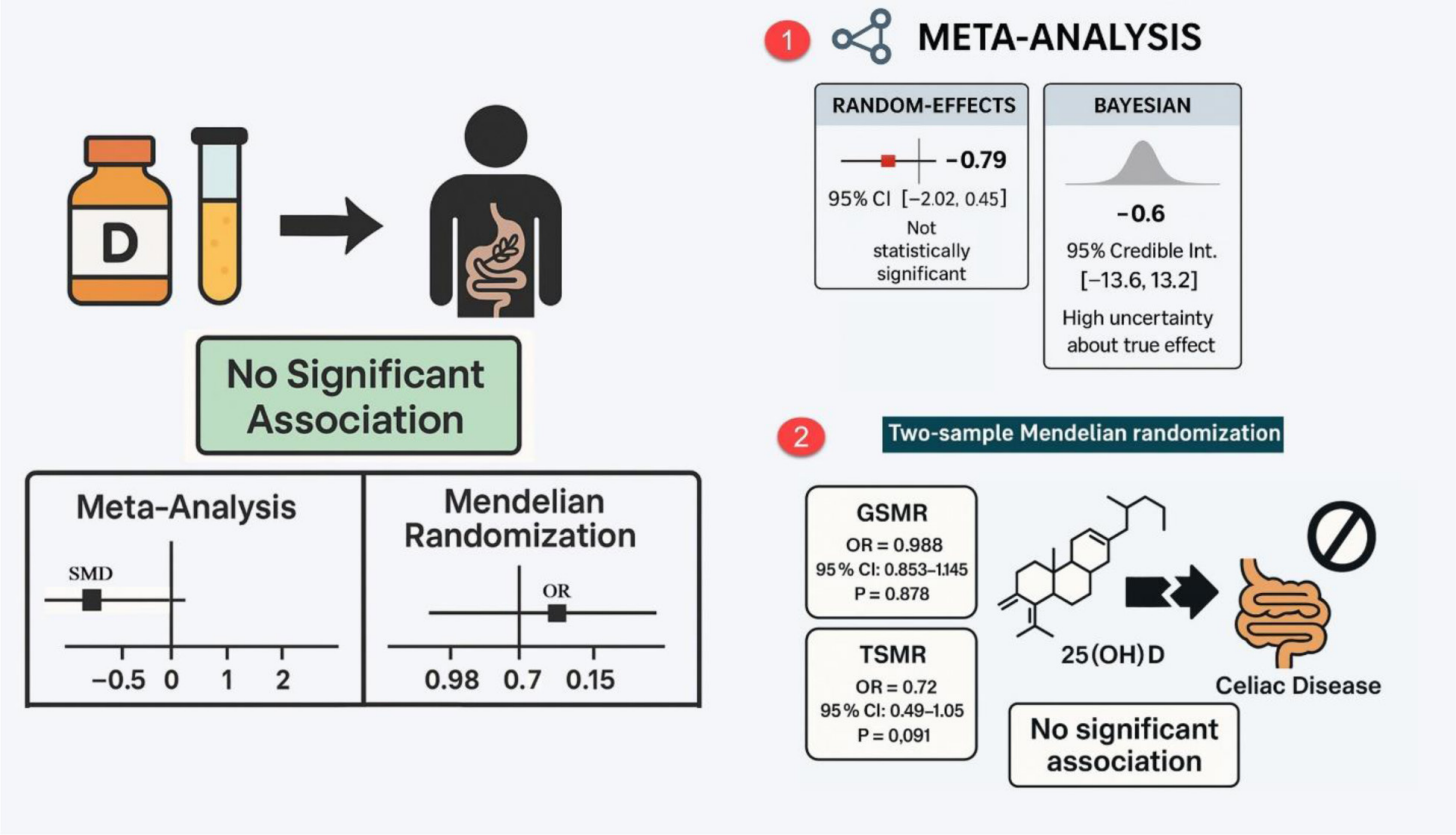
Outlines the 2-part study design for investigating the link between serum 25-hydroxyvitamin D levels and celiac disease. The research consisted of (1) a systematic review with meta-analysis (using both random-effects and Bayesian models), followed by (2) a 2-sample Mendelian randomization analysis. GSMR = Generalized Summary-data-based Mendelian Randomization, TSMR = Two-sample Mendelian Randomization.

We employed 2 different study approaches, as shown in Figure 1. First, we performed a meta-analysis of observational studies to evaluate the association between 25(OH)D and CD. Second, we conducted a MR analysis using genetically determined 25(OH)D-related variants, which mimic the randomized assignment of clinical trials, to assess the long-term influence of 25(OH)D on site-specific disease.

### 2.1. Search strategy and selection criteria

**Figure 2.**
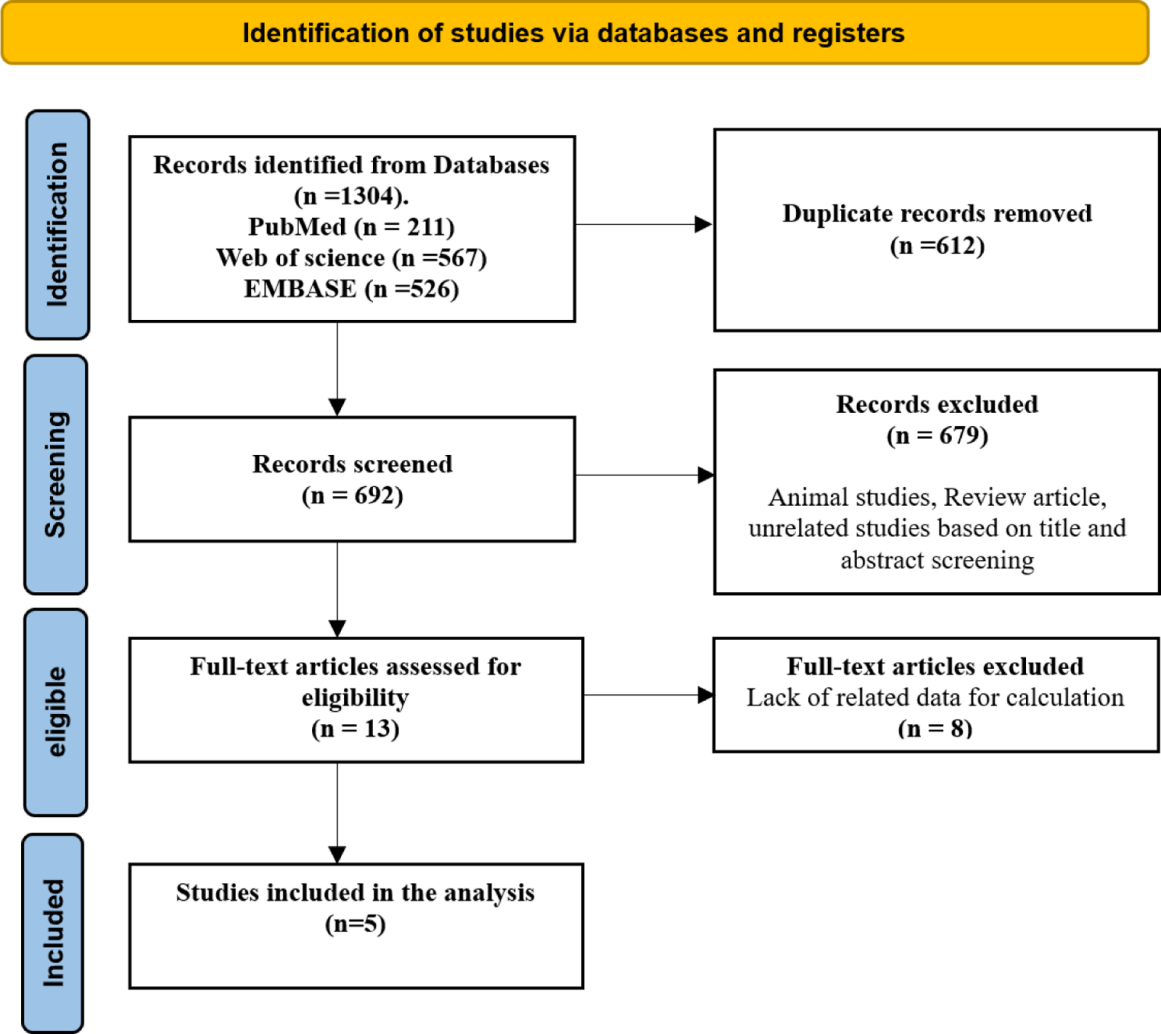
PRISMA flow diagram for Meta-analysis. This plot is a visual representation of the research selection process. PRISMA = Preferred Reporting Items for Systematic Reviews and Meta-Analyses.

We performed a comprehensive literature search in the PubMed, Embase, and Web of Science databases from January 1, 2000 to September 1, 2024, as shown in Figure 2. The following search items were used: ((Coeliac disease) OR (Celiac disease) OR (Celiac sprue) OR (“Gluten Enteropathy”) OR (“Gluten Enteropathies”) OR (“Sprue”) OR (“Gluten-Sensitive Enteropathies”) OR (“Gluten Sensitive Enteropathy”) OR (“Gluten-Sensitive Enteropathy”)) AND ((Vitamin D) OR (25(OH)D_3_) OR (Cholecalciferol) OR (25-Hydroxyvitamin D) OR (Hydroxycholecalciferols) OR (Ergocalciferols) OR (Dihydrotachysterol)) AND ((children) OR (adolescent) OR (pediatric AND (“Gluten Enteropathy” OR “Gluten Enteropathies” OR “Sprue” OR “Gluten-Sensitive Enteropathies” OR “Gluten Sensitive Enteropathy” OR “Gluten-Sensitive Enteropathy”).

Studies to be included needed to European ancestry; pediatric CD; harmonious definition and diagnosis of CD; and keeping data for Vitamin D. Studies that satisfied any of the following criteria were excluded: nonhuman studies; conference abstracts, editorials, or reviews; and full text was not available. If more than 1 study declared the same outcome, we retained the 1 with the most participants. The primary form of Vitamin D measured is the 25(OH)D3 level, which serves as the standard indicator of Vitamin D levels in the body. One unit of 25(OH)D3 is defined as equal to 1 ng/mL, which corresponds to 2.5 nmol/L. Vitamin D levels are typically categorized as follows: Normal: 70 nmol/L or higher. Insufficient: <70 nmol/L. Deficient: <50 nmol/L.

#### 2.1.1. Article assessment

The risk of bias and quality of the included studies were evaluated using the STROBE checklist.

#### 2.1.2. Meta-analysis approach

The meta package^[18]^ in R (version 4.4.2) was employed to conduct the meta-analysis using the standardized mean difference. Statistical heterogeneity was evaluated using Cochran’s *Q* test and the *I*^2^ statistic. Pooled estimates were calculated using a fixed-effects model (Mantel–Haenszel, M–H) if *I*^2^ ≤ 50% and *P* > .05, or a random-effects model (M–H heterogeneity) if *I*^2^ > 50% and *P* ≤ .05. A funnel plot, Begg’s rank test, and Egger’s regression test were used to assess potential publication bias. Galbraith plot and a leave-one-out analysis were performed to evaluate each study’s effect on the overall effect size, identify influential studies, and detect potential outliers.

Additionally, a Bayesian approach was implemented by using a combination of a normal prior for the overall effect size (mean = 50, sd = 50) and a half-Cauchy prior for heterogeneity (scale = 10) in the bayesmeta package^[19]^ (Fig. S1, Supplemental Digital Content 1). Effect sizes and standard errors were derived using the escalc function in the metafor package.^[20]^

### 2.2. Generalized Summary-data-based Mendelian Randomization (GSMR)

GSMR is an advanced approach in MR that is a causal inference technique leveraging genetic variants as IVs.^[21,22]^ GSMR incorporates all genome-wide significant SNPs (*P* < 5.0 × 10^−8^) associated with the exposure, ensuring they are present in the exposure, outcome, and LD reference datasets. Before analysis, GSMR checks for allele frequency discrepancies between the GWAS summary data and the LD reference sample, excluding SNPs with differences exceeding a threshold (default: 0.2). Additionally, clumping is applied (*r*^2^ < 0.05) to select independent IVs. To address horizontal pleiotropy, GSMR employs HEIDI-outlier analysis, removing SNPs showing pleiotropic effects (*P*_HEIDI_ < .01). The method also minimizes chance correlations among SNP instruments, with a default false discovery rate threshold of 0.05 for significance. Furthermore, a two-sample Mendelian randomization (TSMR) analysis was conducted to further explore the relationship.

### 2.3. Two-sample Mendelian randomization (TSMR)

This research report adheres to the STROBE-MR guidelines for observational studies employing MR.^[23]^

#### 2.3.1. Study design

MR has emerged as a method in epidemiology for investigating the causal relationships between risk factors and diseases. MR uses genetic variants that are robustly associated with specific exposures – such as biomarkers or environmental factors – as instrumental variables to infer causality.^[24]^ This approach is particularly valuable because it addresses common limitations in observational studies, such as confounding and reverse causation.^[25]^ Unlike observational studies, where the temporal relationship between exposure and outcome can be ambiguous, MR leverages the fact that genetic variants are fixed at conception and remain constant throughout life.^[26]^ As a result, these variants are not influenced by the disease process, providing a more reliable estimate of the causal effect. Moreover, because genetic variants reflect lifelong exposure, MR can offer insights into the long-term effects of altering specific biomarkers, such as vitamin D levels, on disease risk.^[27]^

In this study, we employed a TSMR approach to explore the potential causal effect of serum 25-hydroxyvitamin D [25(OH)D] concentrations (exposure) on the risk of developing CD (outcome) in individuals of European ancestry (Fig. S2, Supplemental Digital Content 2). By utilizing summary-level data from genome-wide association studies (GWAS), we were able to draw upon large-scale, publicly available datasets that enhance the statistical power of our analysis. This approach allowed us to rigorously test the hypothesis that lower levels of vitamin D may increase the risk of CD.

#### 2.3.2. Data sources

In this study, we employed the datasets available from the IEU Open GWAS Project (https://gwas.mrcieu.ac.uk/datasets/) for 25(OH)D and CD with the specific IDs of “ebi-a-GCST90000618” and “ebi-a-GCST005523,” respectively. To mitigate the confounding factors impacts, we drew upon genetic associations from independent GWAS datasets, all from the same ancestral population. The genetic data for 25(OH)D levels were obtained from a large genome-wide association study involving 4,96,946 individuals, identifying 143 distinct loci across 112 unique 1-Mb regions.^[28]^ For the CD outcome, we utilized 2 datasets comprising 11,812 cases and 229 controls of European descent.^[29]^

#### 2.3.3. Genetic instrument selection

MR relies on 3 key assumptions to ensure valid causal inference. Relevance of Genetic Instruments (Assumption 1): The selected genetic variants must be strongly associated with the exposure (25(OH)D). Typically, SNPs that reach genome-wide significance (*P* < 5 × 10^−8^) in independent studies are used as instrumental variables. To steer clear of weak instrument bias, we calculated the *F*-statistic for each SNP using 2 formulas: $F=\left(\frac{n-k-1}{k}\right)\left(\frac{R{\;}^{2}}{1-R{\;}^{2}}\right)$ (total strength; *R*^2^ shows how much of the variance the SNPs explain, n is the sample size, and k is the number of IVs) and $\;F={\left(\frac{\beta }{SE}\right)}^{2}$ (average strength).^[30]^ If *F* > 10, the instruments are considered strong.^[31]^

Independence from confounders (Assumption 2): The genetic instruments should not be associated with any confounding factors that influence both the exposure and outcome. Although this assumption cannot be definitively proven, researchers can assess potential violations by examining SNP associations with known confounders. To ensure that the SNPs we selected weren’t related to each other, we performed linkage disequilibrium (*r*^2^ < 0.001 with any other associated SNP within 10 Megabase). From the 1000 Genomes Project reference panel, we identified proxy SNPs that were highly correlated (*r*^2^ > 0.8) with the primary variants of interest.

Exclusion restriction (Assumption 3): The SNPs should affect CD risk only through 25(OH)D (no direct or alternative pathways). Violations of this assumption, known as horizontal pleiotropy, occur when an SNP influences multiple traits independently. While this assumption cannot be fully verified, several robust MR methods can detect and adjust for pleiotropy, providing more reliable causal estimates even if some violations exist. We excluded palindromic SNPs from our study when we couldn’t determine the effect allele due to their symmetry.^[32]^ This occurred if their minor allele frequencies were above 0.42.^[33]^ This step ensured that the effect alleles for SNPs were consistently assigned between exposure and outcome, aligning the SNP effects on exposure with those on the outcome.^[34]^ To avoid potential bias from rare alleles that could introduce statistical noise, we removed SNPs with minor allele frequencies below 0.01.^[35]^ Next, Steiger filtering was performed to exclude SNPs that exhibited a stronger association with the outcome than with the exposure, ensuring the removal of potentially invalid instrumental variables.

#### 2.3.4. Two-sample Mendelian randomization analysis

The main approach used in the univariate MR analysis was the inverse variance weighted (IVW) method, implemented within a random-effects model.^[36]^ Given that IVW can be influenced by pleiotropy or invalid instrument bias, multiple methods – such as MR-Egger, weighted median, simple mode, and weighted mode analyses – were applied to explore causal relationships.^[37]^ This diverse set of techniques ensured a thorough evaluation of potential causal associations. To further enhance the robustness of our findings, additional methods were employed, including Maximum Likelihood, Robust Adjusted Profile Score (RAPS), MR-Least Absolute Shrinkage and Selection Operator, constrained maximum likelihood, mode-based estimation, debiased IVW, contamination mixture, and mixture models.^[38-40]^ These complementary approaches helped ensure the reliability of the results. Additionally, the Steiger test was conducted to confirm the correct directionality of the causal relationship between the variables under study.^[41]^

To validate the findings, several sensitivity analyses were carried out. First, Cochran’s *Q* test and the *I*^2^ statistic (with 95% confidence intervals) were used to assess heterogeneity in MR-inverse-variance weighted analyses, while Rucker’s *Q* statistic was applied for MR-Egger.^[42]^ A *P*-value > .05 indicated no significant heterogeneity. Second, the I^2^GX statistic was calculated to evaluate potential violations of the NOME (No Measurement Error) assumption.^[43]^ Third, horizontal pleiotropy was examined using the MR-Egger intercept and MR-PRESSO global tests.^[44]^ To detect SNPs with potential pleiotropic effects, both MR-PRESSO and RadialMR were utilized.^[45,46]^ Cook’s distance and Studentized residuals were also analyzed to identify outliers or influential SNPs.^[46]^ If specific SNPs were flagged as potentially contributing to heterogeneity or bias, causal estimates were recalculated after their exclusion. Fourth, a leave-one-out sensitivity analysis was performed to determine whether any single SNP disproportionately affected the results.^[47]^ Fifth, funnel plots were generated to visually inspect for signs of pleiotropy, further ensuring the robustness of the findings.^[44]^

All statistical analyses were conducted using RStudio (version 2024.12.1 + 563), leveraging packages such as “TwoSampleMR,” “MendelianRandomization,” “MR-PRESSO,” “RadialMR,” “MRMix,” “mr.raps,” and “GMRP.” The “mrrobust” package was also used in STATA (version 17) to complement the analysis. Results were reported as odds ratios (OR) and β coefficients (β), along with their respective 95% confidence intervals. A *P*-value threshold of < .05 was considered indicative of suggestive evidence for a relationship.

#### 2.3.5. MR-Cluster analysis

Subsequently, MR-Cluster was employed to assess heterogeneity in the estimated association effects of 25(OH)D on CD. This method accounts for variations in uncertainty across association estimates and groups genetic variants with similar effects into distinct clusters. For the clustering analysis, 13 genetic variants were included based on their association estimates for 25(OH)D and CD to understand whether groups of genetic variants collectively influenced traits. Only clusters containing at least 4 variants with a conditional probability of≥0.8 were retained. The analysis was conducted using the “mrcluster” R package in the MR results.^[48]^

## 3. Results

### 3.1. Meta-analysis

A comprehensive search of 1304 potentially unique references was conducted. Out of these, 5 studies fulfilled the established criteria for inclusion.^[49–53]^ The 5 studies encompassed a total of 832 participants, consisting of 377 individuals with CD and 455 non-CD participants.

There was evidence of high heterogeneity among the selected 5 studies (*I*^2^ = 96.5%, 95% CI: 94.1–97.9; *Q*_Heterogeneity_ = 114.33, *P* ≤ .001). The pooled estimate of standardized mean difference based on a random-effects model was −0.79 (95% CI: −2.02, 0.45), and was not statistically significant (*T*_Statistic_ = −1.77, *P* = .152). No evidence of publication bias was found (Begg’s test: *P* = .071, Egger’s test: *P* = .435). This finding remained consistent after several sensitivity analyses (Figs. S3–S6, Supplemental Digital Content 3), including a Galbraith plot (Fig. S3, Supplemental Digital Content 3), a leave-one-out analysis (Fig. S4, Supplemental Digital Content 4), and funnel plots for publication bias (Figs. S5 and S6, Supplemental Digital Content 5).

**Figure 3.**
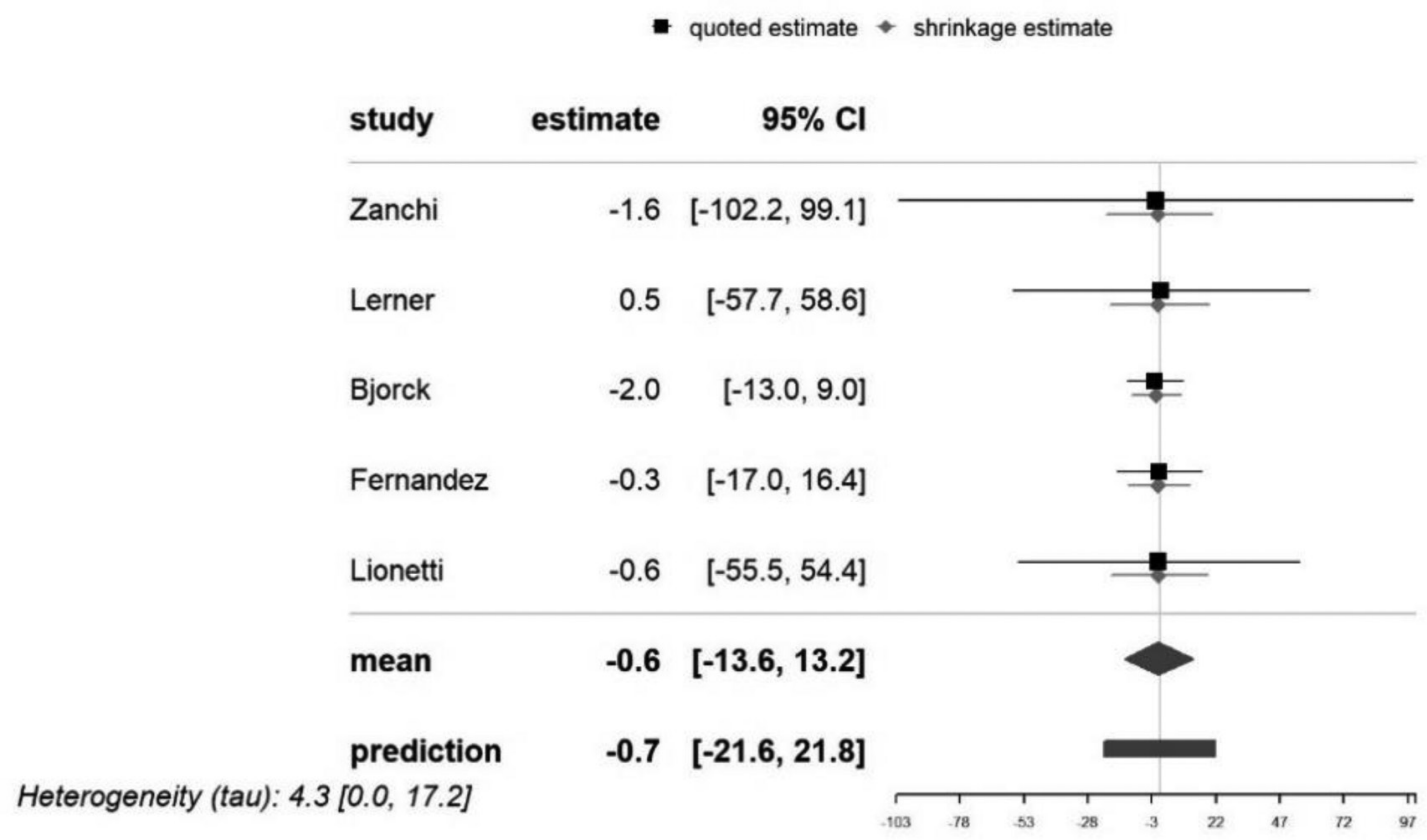
The forest plot displays 5 individual estimates (represented by the 5 black horizontal lines) along with their 95% confidence intervals. Quoted estimates reflect individual study findings, while shrinkage estimates provide a more comprehensive view of the overall effect, accounting for potential heterogeneity. At the bottom of the plot, there are 2 additional elements. A diamond shape centered at the posterior median represents the combined estimate. This diamond spans the 95% credible interval for the overall effect. A horizontal bar at the bottom shows the 95% prediction interval. This interval predicts where a new study might fall if added to the meta-analysis. CI = confidence interval.

**Figure 4.**
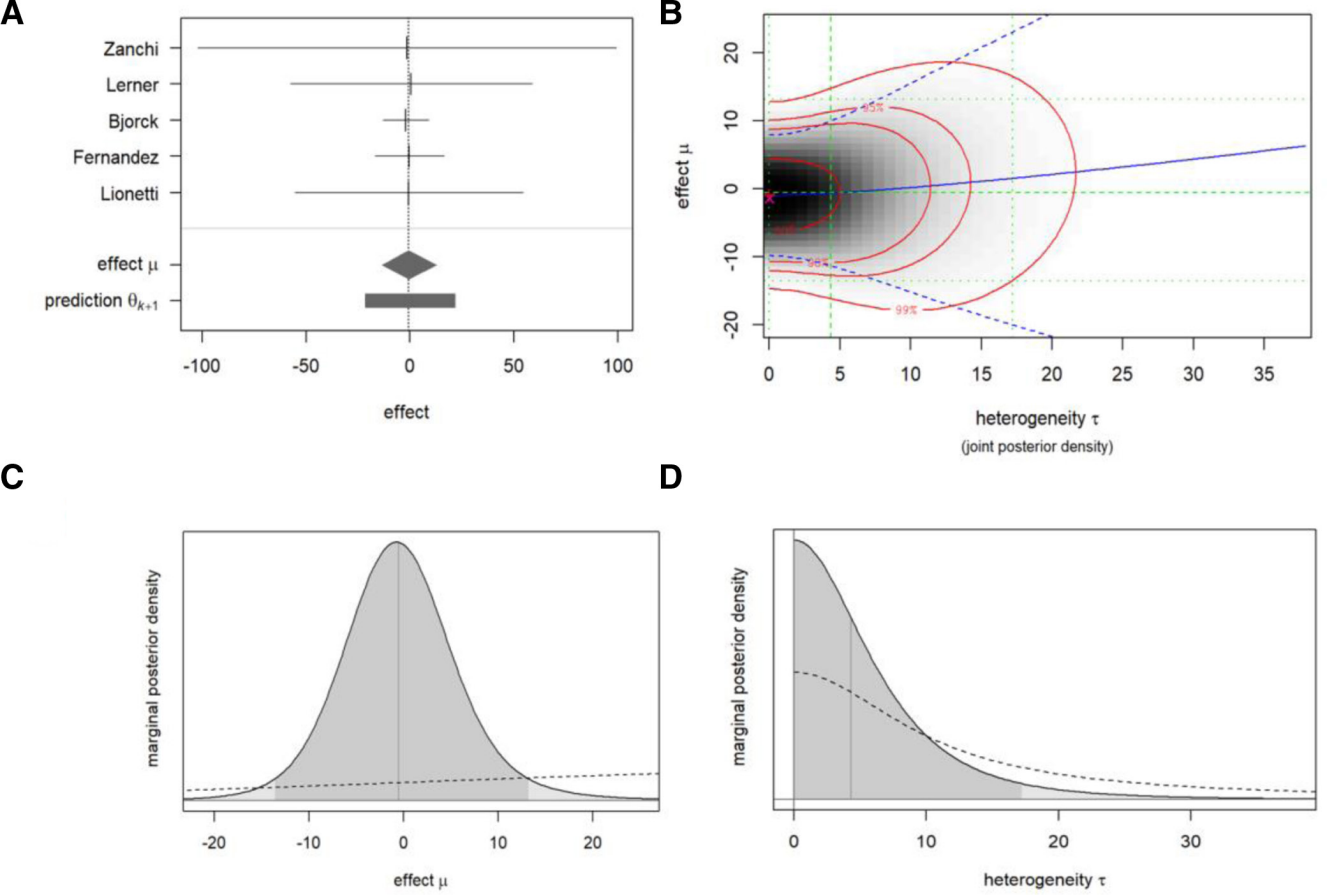
Posterior distribution. (A) A simple forest plot showed the 5 estimates along with the combined estimate (diamond) and prediction interval (bar). (B) Illustrated the joint posterior density of both parameters. Darker shading indicated higher posterior density values. The red contour lines show (approximate) 90%, 95%, and 99% confidence regions for the joint distribution. The solid blue line traces the conditional posterior expectation value, and the dashed lines enclose the corresponding 95% interval as a function of heterogeneity. The green lines indicate marginal posterior median and 95% intervals for both parameters. (C, D) Showed the marginal density functions.

The Bayesian meta-analysis (Fig. 3) further supported these findings, displaying a 95% credible interval that ranged from −13.6 to 13.2, indicating considerable uncertainty around the effect sizes. Therefore, the result of the Bayesian meta-analysis is the same as the results of the previous meta-analysis. Posterior distribution supported the conclusion that a general trend exists, as shown in Figure 4.

### 3.2. Generalized Summary-data-based MR

The GSMR analysis showed no significant link between 25(OH)D levels and CD (OR = 0.988; 95% CI: 0.853–1.145; *P* = .878; SNPs = 64). This association was further investigated using a TSMR design.

### 3.3. Two-sample Mendelian randomization

Initially, 117 SNPs were extracted from Vitamin D as instrumental variables, considering a significance threshold of *P* < 5 × 10^−8^ and Linkage Disequilibrium (LD) clumping. After the harmonization process, a total of 22 SNPs had outcome data available for further analysis of the causal relationships between Vitamin D and CD. Two palindromic SNPs (rs2511279 and rs8107974) were removed, and the outcome data were updated (Supplementary data, Supplemental Digital Content 6). Since the *F*-statistics for the instruments ranged from 30.06 to 1474.55, the weak instrumental bias can be statistically ignored (Supplementary data, Supplemental Digital Content 6).

**Figure 5.**
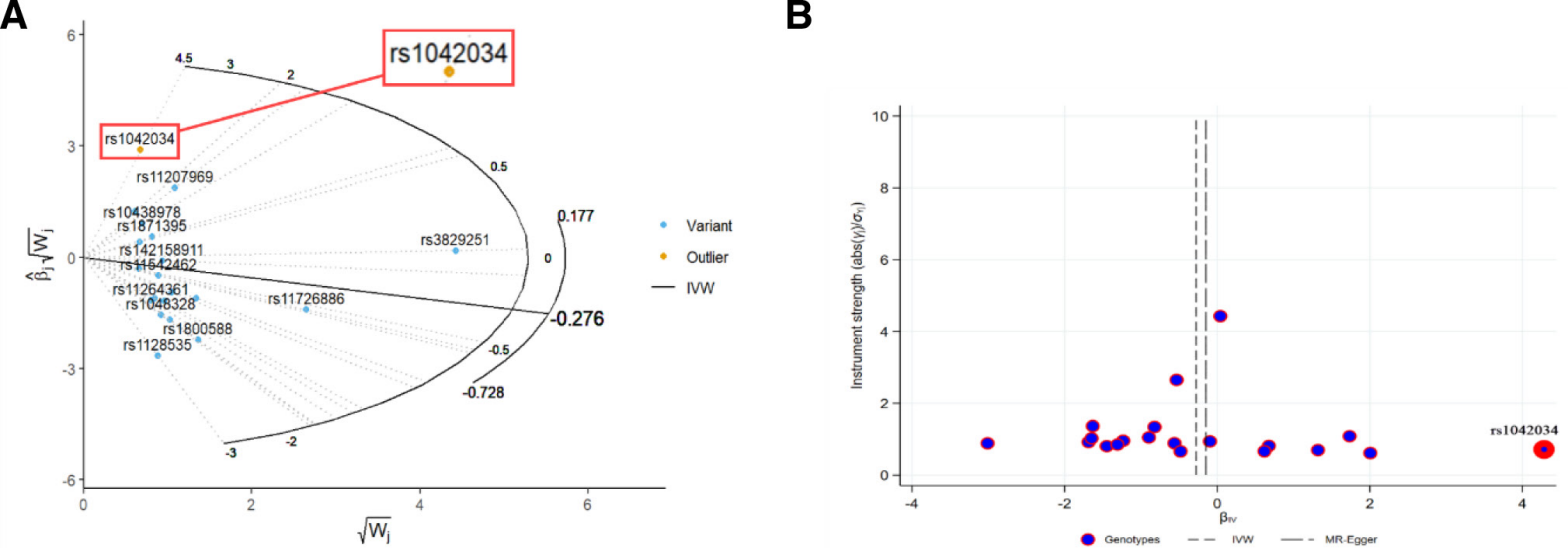
(A) Radial plot. It provides insights into heterogeneity and outliers. The orange dot represents an outlier SNP (here rs1042034 in the APOB gene). Each blue dot represents an SNP. The black curve represents the estimated IVW regression fit using the radial method. (B) Funnel plot. The rs1042034 (APOB gene) is marked as a potential influential point. SNP = single nucleotide polymorphism.

**Figure 6.**
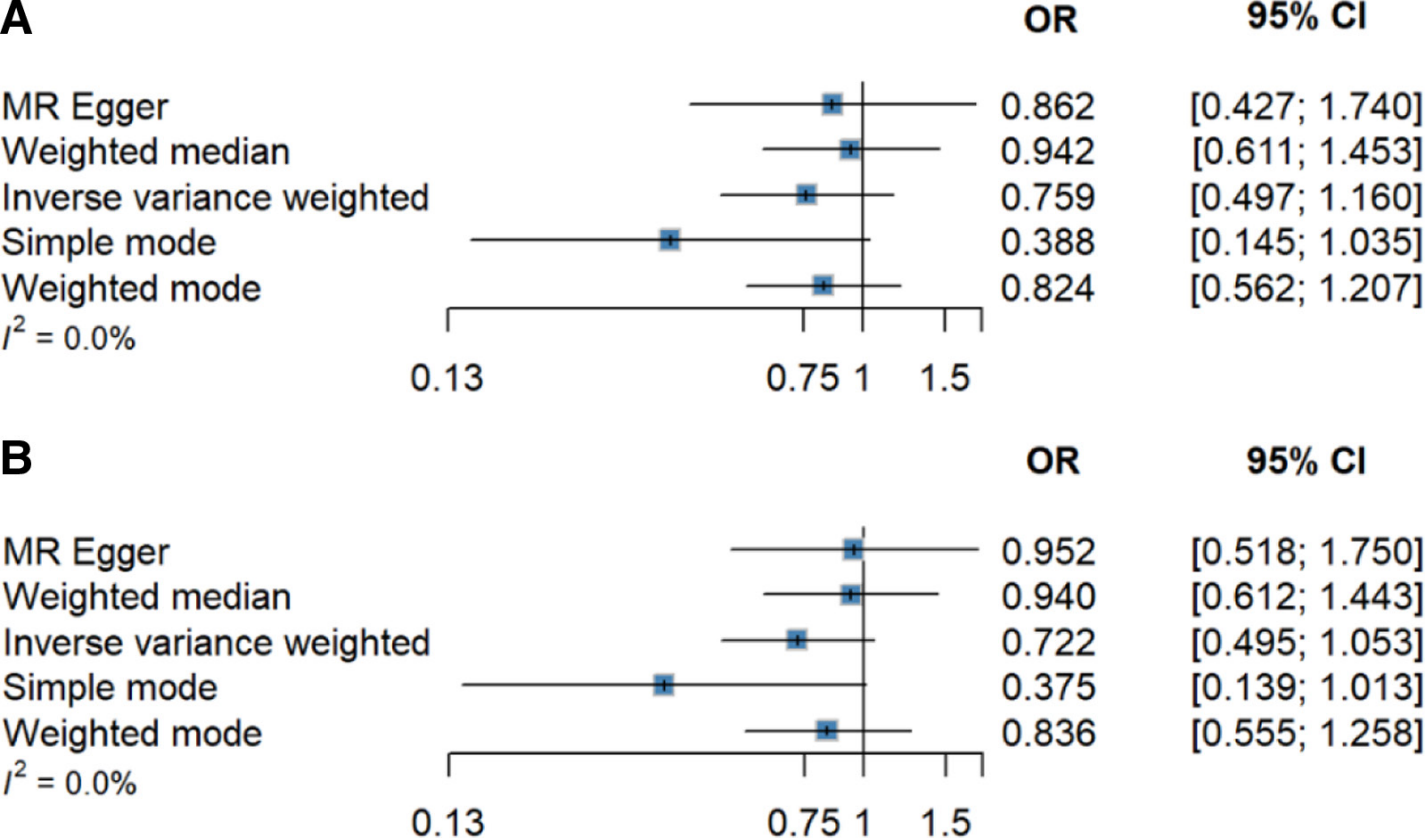
Shows the analysis (A) including and (B) excluding the potential SNP (rs1042034). MR-PRESSO, MR-radial, Cook’s distance, and Studentized residuals were used to determine whether specific SNPs were identified as outliers or influential points. CI = confidence interval, OR = odds ratio, SNP = single nucleotide polymorphism.

To evaluate homogeneity and pleiotropy, significant heterogeneity was detected using both IVW (*Q* = 37.37, *Q*_pvalue_ = 0.007; *I*^2^ = 49.2%) and MR-Egger (*Q* = 36.95, *Q*_pvalue_ = 0.005). However, the pleiotropy test suggested no evidence of pleiotropy (Egger intercept = -0.005, *P*-value = .659). To further investigate potential influential point(s), additional methods including MR-PRESSO and Radial-MR, and Cook’s distance and Studentized residuals were employed. MR-PRESSO, RadialMR, and Cook’s distance identified the SNP “rs1042034” as an outlier (Fig. 5). Removing the “rs17430818” effectively improved the issue of heterogeneity (*Q*_IVW_ = 27.74, *P* = .066, *I*^2^ = 35.1%; *Q*_Egger_ = 25.80, *P* = .078, *I*^2^ = 34.1%; *I*^2^_GX_ = 98.41%), as shown in Figure 6. The β of serum 25 hydroxyvitamin D concentrations regressed on the β of CD was illustrated to demonstrate the relationship of the undefined causal variables to CD (Fig. S7, Supplemental Digital Content 7), which visualizes the slope estimated by the various MR methods.

**Figure 7.**
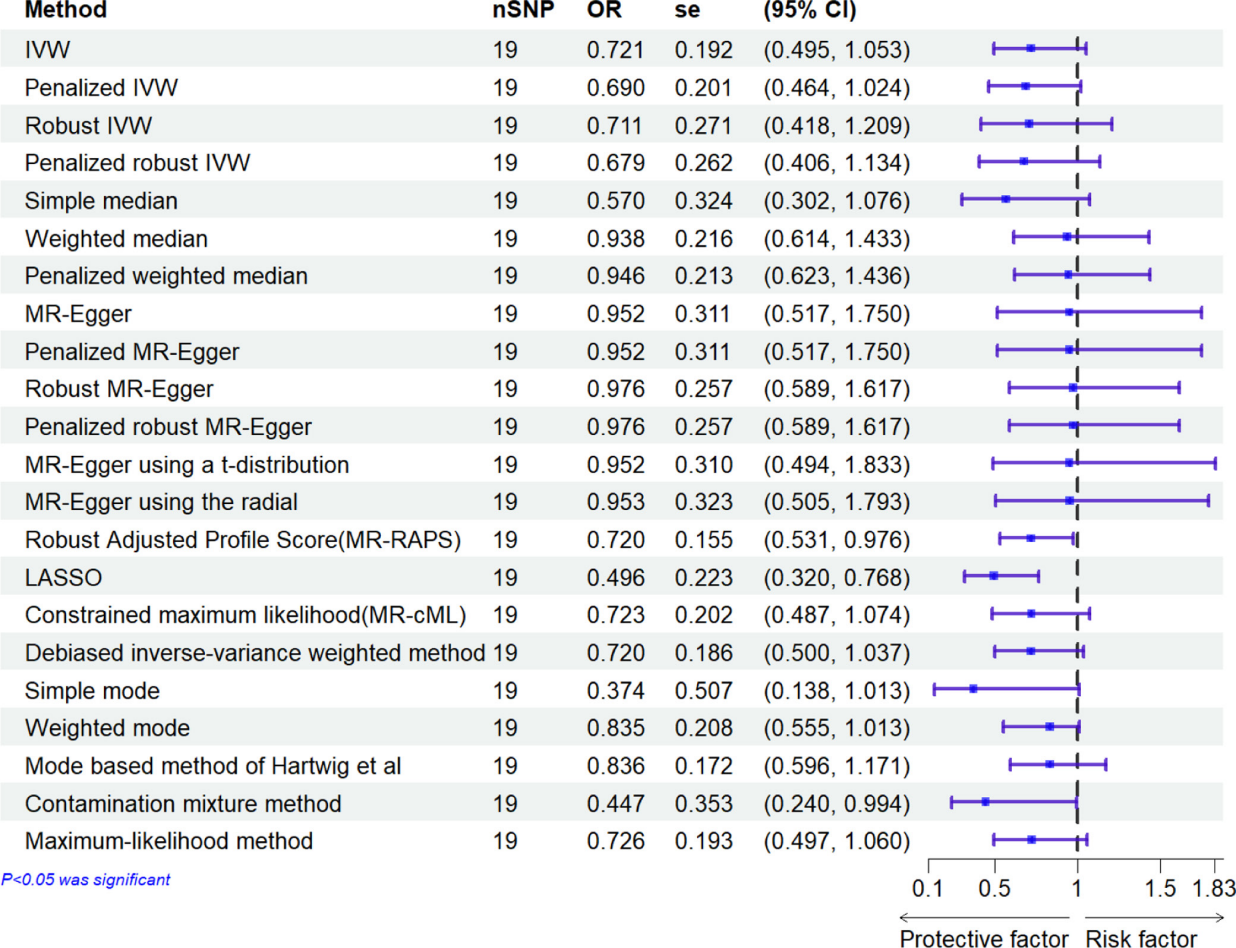
Forest plot. Application of multiple MR methods to examine the relationship between serum 25-hydroxyvitamin D and celiac disease. CI = confidence interval, IVW = inverse variance-weighted, nSNP = number of single nucleotide polymorphism, OR = odds ratio, SE = standard error.

The association between 25-hydroxyvitamin D levels and CD was not statistically significant (OR_IVW_ = 0.72, 95% CI: 0.49–1.05, *P*-value = 0.091). The main finding was confirmed by several methods, as shown in Figure 7.

**Figure 8.**
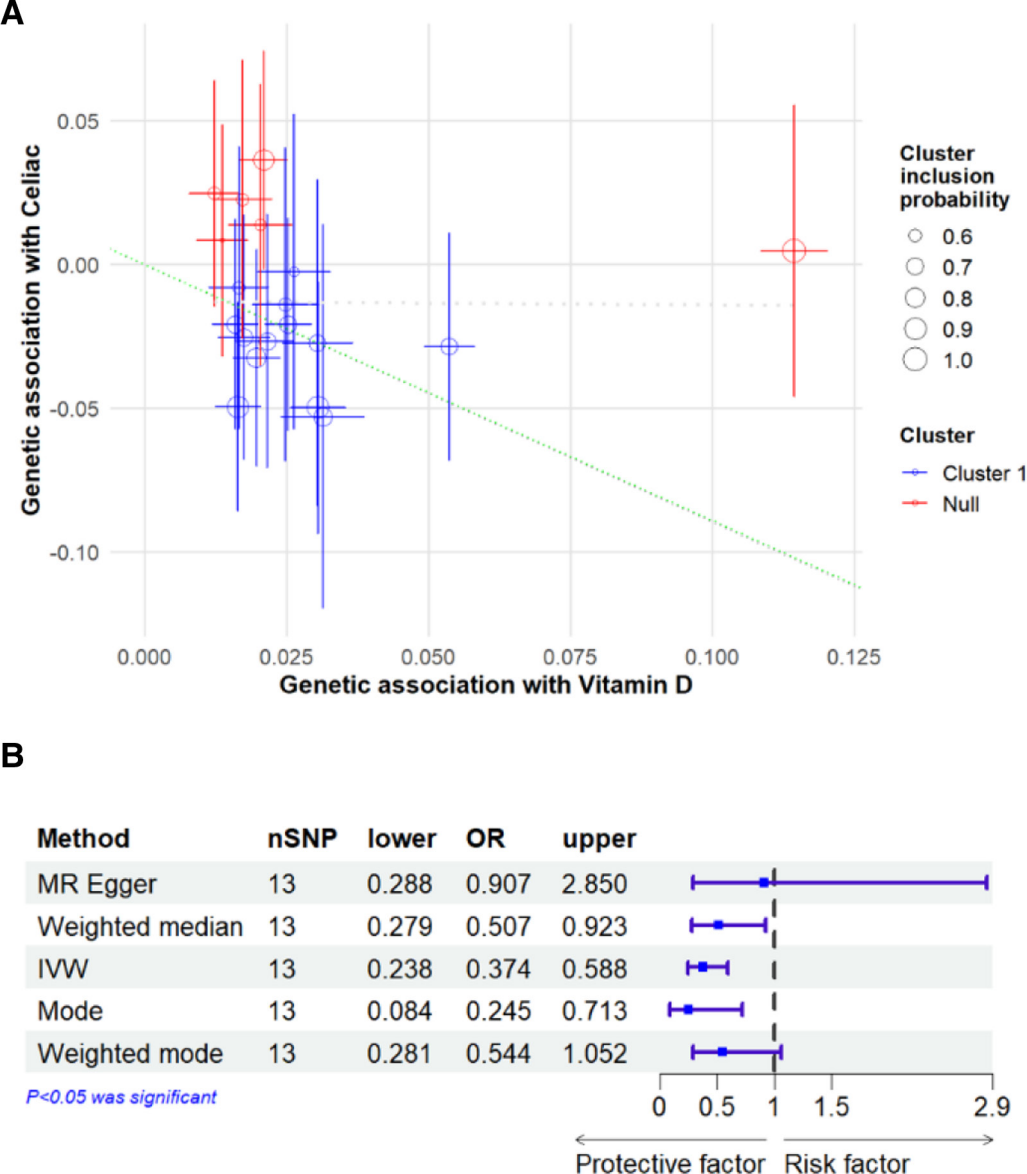
(A) A scatter plot illustrating the 2-stage regression reveals clustered genetic associations between serum 25-hydroxyvitamin D concentrations and celiac disease. Each point represents a genetic variant. The black-colored cluster (Cluster 1) contains variants that show stronger shared effects between the 2 traits. (B) Forest plot using cluster 1. IVW = inverse variance-weighted, MR = Mendelian randomization, nSNP = number of single nucleotide polymorphism, OR = odds ratio.

Moreover, the data points do not show significant differences or reveal distinct genetic association patterns for vitamin D and CD (Fig. 8). This suggests the existence of 1 cluster or grouping within the dataset. Some evidence suggests an inverse relationship between vitamin D levels and the risk of CD, although there is some heterogeneity. The null cluster (pink) represents variants that do not significantly influence this association. In contrast, the presence of high-probability variants (large circles) in Cluster 1 indicates that certain genetic variants play a significant role in driving the observed effect.

#### 3.3.1. Sensitivity analyses

The results of Cochran’s *Q* test showed no significant heterogeneity after removing the outlier SNP. Furthermore, no evidence of directional horizontal pleiotropy in CD was found, as supported by the MR-Egger regression intercept analysis. To detect potential outlier SNPs, we employed multiple methods, including the MR-PRESSO test, Radial-MR, Cook’s distance, and Studentized residuals. Scatterplots (Fig. S8, Supplemental Digital Content 8), leave-one-out analyses (Fig. S9, Supplemental Digital Content 9), and funnel plots (Fig. S10, Supplemental Digital Content 10) were visually examined to evaluate the influence of outliers. The relationship between serum 25-hydroxyvitamin D levels and CD is illustrated in a forest plot (Fig. S11, Supplemental Digital Content 11). Weak instrumental bias was assessed using the *F*-statistic (Supplementary data, Supplemental Digital Content 6). The directionality of the causal relationship for CD was confirmed using the MR Steiger test, which produced a nonsignificant result (*P* > .05). We also investigated the densities of IV estimates across varying phi parameter values, as presented in Figure S12, Supplemental Digital Content 12. A comprehensive summary of all analytical steps is provided in Figure S13, Supplemental Digital Content 13.

## 4. Discussion

The literature presents inconsistent findings concerning the role of VitD deficiency in the development of CD. Contrary to the results of the present analysis, a nationwide study conducted in Turkey involving 6717 children and adolescents with diagnosed CD and an equal number of controls unearthed that the rate of VitD deficiency was notably accentuated in the CD group, with significantly reduced 25(OH)D levels observed in these individuals.^[6]^

In a similar vein, Marino and colleagues examined 25(OH)D levels at the point of diagnosis for CD and type 1 diabetes in children, uncovering decreased plasma 25(OH)D concentrations across all these conditions. Interestingly, they discovered that none of the children with CD exhibited severe VitD deficiency, whereas both type 1 diabetes and the combination of type 1 diabetes and CD were linked to significant VitD deficiency.^[54]^ A recent meta-analysis conducted by Sun et al involving pediatric patients with CD established that the prevalence of VitD deficiencies in CD cases was significantly greater than in control groups, with the affected individuals exhibiting notably lower VitD levels.^[2]^ Another meta-analysis focusing on 25(OH)D status in adults and children with CD underlined markedly reduced VitD levels in affected patients, which normalized following treatment.^[14]^ On the contrary, some research has shown that VitD concentrations do not differ significantly between individuals with CD and control subjects. In line with our findings, Lerner and colleagues conducted a comparison of 25(OH)D status among pediatric patients with CD and those without, as well as their parents and adult cases of CD. They reported that none of the children with CD exhibited VitD deficiencies, whereas notable deficiencies were found in the adults with the condition.^[50]^ The rationale for this finding was explained as being related to the increased VitD intake among children with CD, which was facilitated by regular supplementation in the early months of life, along with augmented sun exposure and adherence to a GFD during early life. Interestingly, they found that VitD deficiency was influenced by age; however, this did not appear to correlate with the extent of small bowel damage in CD.^[50]^ Two retrospective studies yielded comparable findings, indicating no significant differences in 25(OH)D status between children with CD and those without.^[13,55]^

To assess the role of VitD in the onset of CD, researchers concentrated on VitD levels not only following the diagnosis but also prior to it, particularly during pregnancy and the early years of life.^[56]^ In alignment with our findings, a multi-site diabetes investigation explored the impact of prenatal supplementation on the risk of CD and presented no significant link between prenatal VitD supplementation and the likelihood of developing CD.^[57]^ Likewise, a cohort study conducted in Norway yielded no connection between supplementation during the first 18 months of life and the risk of CD, nor was there an association between prenatal supplementation and CD risk.^[17]^ A nested case-control study selected from a birth cohort of 8676 genetically susceptible children, who were prospectively tracked in the multinational Environmental Determinants of Diabetes in the Young (TEDDY) initiative, found no notable correlation between 25(OH)D levels and susceptibility to CD during early infancy and childhood. Meanwhile, in the classification of 25(OH)D levels, an augmented risk of CD autoimmunity was observed in genetically predisposed children with concentrations below 30 nmol/L and above 75 nmol/L during early infancy, which aligns with the results of our investigation.^[58]^

In individuals with managed CD, the necessity for VitD supplementation appears to be less critical. In the meta-analysis by Lu et al, which was mentioned earlier, 25(OH)D levels in individuals with CD were found to be comparable to those of healthy controls following treatment.^[14]^ However, dietary increases alone may not be adequate to restore serum VitD levels to normal. Children with CD who had VitD deficiency and were treated with a GFD plus a weekly dose of 60,000 IU of VitD for the initial 3 months exhibited a notable rise in their serum VitD levels; nevertheless, none achieved normal VitD levels.^[59]^

The relationship between VitD and CD can be viewed through a multifaceted lens arising from the intricate interplay of genetic, immune, and environmental factors.^[14]^ The potential role of VitD in the pathogenesis of CD may be understood through 2 principal biological mechanisms. Firstly, VitD is known to regulate immune function, notably by influencing the activity and differentiation of T-lymphocytes and modulating dendritic cell responses, both of which are central to the immune dysregulation observed in CD. Secondly, VitD may affect intestinal barrier integrity by altering cytokine profiles and influencing the zonulin-mediated pathways that govern epithelial permeability.^[54]^

Immune system components, including dendritic cells and B- and T-lymphocytes, are modulated by the regulatory actions of VitD through the VDR. This receptor is essential for the biological activities of VitD, and these immune cells also have the capacity to produce active VitD locally.^[60]^ Research has focused on the expression and kinetics of VitD-associated genes in stimulated human T-lymphocytes. A study indicated that 1,25(OH)_2_D_3_ robustly activates the VDR pathway, but this activation is predominantly observed in T-lymphocytes that have high VDR expression levels. These results indicate that increased VDR signaling is associated with a more pronounced suppression of cytokines, identifying T-lymphocytes as direct targets of 1,25(OH)_2_D_3_ within the immune system.^[61]^ In addition, a meta-analysis examining the susceptibility associated with genetic variants of the VDR identified a relationship between the decreased serum 25(OH)D levels and the Fok1 polymorphism (rs2228570, *C* > *T*) of the VDR gene in relation to CD.^[3]^

Gluten is composed of 2 main categories of proteins: glutenins and prolamins, with prolamins encompassing gliadin, secalin, and hordein.^[62]^ The process of gluten breakdown yields pepsin-trypsin-resistant gliadin (PT-G), an undigested component of gliadinthat plays a dominant role in the pathogenesis of CD by hampering intercellular tight junctions. Tight junctions orchestrate the selective barrier function of the intestinal mucosa.^[62]^ Zonulin, analogous to the prokaryotic zonula occludens toxin, functions as an enterotoxin in primates. It is released by intestinal epithelial cells in response to nutritional factors or microbiota triggers and has been demonstrated to be a key regulator of tight junctions and gut permeability.^[63]^ Drago and colleagues reported that exposing duodenal biopsies from subjects with CD to PT-G triggers a prolonged release of zonulin, which is thought to be a precursor of haptoglobin 2.^[64]^ It has been postulated that the heightened intestinal permeability resulting from gluten ingestion and the subsequent secretion of zonulin allow gliadin to penetrate the submucosa. Gliadin activates macrophages via a MyD88-dependent mechanism, fostering a Th1 cytokine pool characterized by increased levels of TNF-α, IL-12 p70, and IL-15. This process occurs through the upregulation of genes associated with inflammation and the enhanced release of cytokines.^[65]^

In this context, the significance of vitD is particularly noteworthy. The impact of 1,25(OH)_2_D_3_ on tight junction injuries caused by PT-G has been studied.^[62,66]^ The most significant impact of 1,25(OH)_2_D_3_ in alleviating monolayer barrier damage was observed at a concentration of 10^−8^ M, which proved optimal for enhancing tight junction protein expression. A concentration of 10^−8^ M 1,25(OH)_2_D_3_ exerts the strongest influence in reducing zonulin release, and at this same level, it also inhibits the expression of MyD88, a molecule that triggers zonulin secretion.^[66]^

A growing body of experimental evidence postulates the existence of a bidirectional interaction between CD and vitD status.^[67]^ Sufficient vitD levels enhance the body’s ability to tolerate inflammatory responses and may offer protection against the development of CD.^[68]^ Building on this rationale, inadequate vitD levels could be a contributing factor in the development of CD. Clinical observations suggest that, in some patients diagnosed with CD, particularly those lacking typical gastrointestinal symptoms, vitD deficiency may be the only identifiable issue. Furthermore, decreased vitD concentrations have been observed during the initial phases of the disease.^[1,4]^ On the other hand, in CD, a combination of hereditary predisposition and interaction with environmental factors contributes to the deterioration of gut barrier functionality. Gluten peptides and gliadins can compromise gut permeability, potentially leading to impaired absorption of vitD and resulting in deficiency.^[62]^ This may account for the diminished vitD levels noted following the development of CD. Moreover, this deficiency in vitD, combined with the dysregulated transport of macromolecules resulting from the “leaky gut,” significantly harms the intestine, creating a feedback loop that perpetuates the inflammatory process.^[62,68]^

The overall null findings from both our MR analysis, leveraging GWAS-derived genetic instruments, and our meta-analysis of observational studies suggest no causal relationship between reduced vitD levels and the development of CD. This convergence across 2 complementary methodological approaches strengthens the validity of our conclusion and reduces the likelihood that unmeasured confounding or reverse causation is responsible for the observed results. Furthermore, the results of sensitivity studies and the use of different MR techniques were consistent, confirming the validity and dependability of the conclusions reached.

The overall null findings from our MR analysis and meta-analysis stand in contrast to a number of observational studies that have reported an association between low vitamin D levels and a higher prevalence of CD.^[2,6]^ We posit that these discrepant results are best explained by fundamental methodological differences between study designs. Observational studies are highly susceptible to confounding by factors such as diet, sunlight exposure, physical activity, and socioeconomic status, all of which influence both vitamin D levels and general health outcomes, potentially creating a spurious association with CD risk. More critically, they cannot reliably establish the direction of causality. Our findings support a model of reverse causation, whereby the intestinal malabsorption and inflammation characteristic of active CD lead to lower serum 25(OH)D concentrations. This is a more biologically plausible explanation than a causal effect of vitamin D on disease onset, as it aligns with the known pathophysiology of CD and the fact that vitamin D levels often normalize after treatment with a gluten-free diet. While our meta-analysis also found no significant difference, its observational nature and high heterogeneity limit its conclusiveness. In contrast, the MR approach, by using genetic variants fixed at conception, is largely immune to reverse causation and confounding, providing a more robust test of a causal hypothesis. Therefore, the discrepancy with earlier literature likely arises not from an error in either set of findings, but from the fact that observational correlations capture a consequence of the disease, while MR investigates its cause. Our study underscores the importance of using genetic methods to triangulate evidence from observational epidemiology to distinguish causal drivers from downstream effects. The discrepancy in reported results may be explained in part by the fact that these characteristics were not controlled for in previous studies.^[4]^

### 4.1. Strengths and limitations

Despite several methodological strengths, including the integration of MR and meta-analysis approaches, this study has important limitations that should be acknowledged. Fundamental presumptions underlie MR, specifically that the chosen genetic variations affect the result only through the exposure of interest.^[69]^ The idea of horizontal pleiotropy cannot be completely ruled out, even though statistical analyses did not show any compelling evidence of pleiotropic bias. Furthermore, both the MR and meta-analytic components were restricted to populations of European ancestry, thereby constraining the external validity of our results. The meta-analysis of observational investigations also encountered challenges, such as significant between-study heterogeneity, diversity in vitD assessment techniques, uneven criteria for diagnosing deficiency, and the possibility of residual confounding. Taken together, these limitations highlight the necessity for more research in more ethnically varied cohorts, as well as the adoption of standardized procedures in future observational studies, to validate and expand our findings.

### 4.2. Clinical implications

Our findings have important implications for clinical practice and research. The absence of a causal effect of genetically determined 25(OH)D on CD risk suggests that population-wide vitamin D supplementation is unlikely to be an effective strategy for the primary prevention of CD. This should help redirect public health efforts towards other modifiable risk factors. However, it is crucial to distinguish between causation and management.^[70–72]^ Our results do not contradict the well-established role of vitamin D in bone metabolism. Therefore, in patients with established CD, vitamin D deficiency – likely resulting from disease-related malabsorption – remains a significant concern for bone health. The clinical guidance to screen for and treat vitamin D deficiency in diagnosed CD patients to prevent osteoporosis and other complications remains entirely valid and is supported by a different body of evidence. The key distinction our study provides is that correcting this deficiency should be viewed as managing a consequence of the disease, not as a treatment targeting the underlying autoimmune pathogenesis.

## 5. Conclusion

In summary, the integration of MR, leveraging GWAS-based genetic instruments, and a comprehensive meta-analysis of observational data revealed no support for a causal relationship between lower vitD levels and the onset of CD. These convergent findings challenge the assumption that vitD deficiency directly contributes to CD pathogenesis and may inform clinical perspectives on supplementation and disease prevention. However, these interpretations should be approached with measured caution, given the methodological constraints inherent to MR analyses and the multifactorial nature of vitD metabolism in relation to immune-mediated disorders such as CD.

## Acknowledgments

We want to acknowledge the participants and investigators who made summary data available.

## Author contributions

**Conceptualization:** Danial Habibi

**Data curation:** Taha Rafiei, Soheil Rahmati, Yasamin Jiani, Danial Habibi

**Formal analysis:** Mahdi Akbarzadeh, Danial Habibi

**Methodology:** Mahdi Akbarzadeh, Danial Habibi

**Software:** Mahdi Akbarzadeh, Alireza Soleymani Taloubaghi

**Writing – original draft:** Mahdi Akbarzadeh, Fahimeh Haghighatdoost, Amirhossein Ataei Kachouei, Danial Habibi

**Writing – review & editing:** Amir Hesam Saeidian, Fereidoun Azizi, Mehdi Hedayati, Maryam S Daneshpour, Mohammad Rafiei, Danial Habibi




























