# Supplementary material for: Genetic predisposition to serum 25 hydroxyvitamin D concentrations does not influence the risk of decreasing celiac disease in European ancestry: Evidence from meta-analysis and Mendelian randomization
Source: Medicine (Baltimore). 2026 Jul 3;105(27):e49587. doi: 10.1097/MD.0000000000049587 (PMC13336962; doi:10.1097/MD.0000000000049587)
Supplement: Supplementary file 1 [file medi-105-e49587-s001.pdf]

Figure S1. Diagram of Mendelian randomization framework in the current paper.

Instrumental variable techniques provide methods for estimating causal effects in observational data without requiring complete knowledge of all confounding factors that influence the exposure-outcome relationship. Mendelian randomisation utilises genetic variants as instrumental variables to investigate causal associations between exposures and outcomes in non-experimental data. For a genetic variant to be considered a valid IV, several key assumptions must be met:

1. The variant should be strongly associated with the exposure and quantifiably so.
2. The variant should not be associated with any confounder of the exposure-outcome association.
3. The variant should not affect the outcome except potentially indirectly through its association with the exposure.

These assumptions are based on Mendel's laws of inheritance, which suggest that genetic variants (SNPs) are unlikely to be associated with confounders or outcomes after they've been determined at conception. This makes them useful as instruments for estimating causal effects in observational studies.

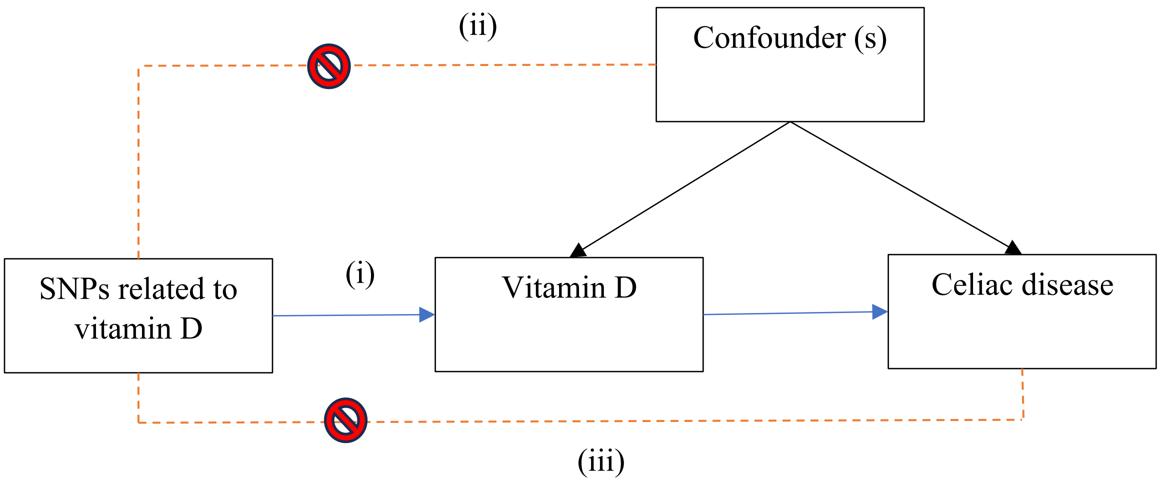

Mendelian randomization design in the current study

Mendelian Randomization was employed to investigate the causal impact of vitamin D on Celiac disease. Considering the fact that all three assumptions of valid instruments are satisfied(1), the most efficient method to estimate the causal effect is the inverse variance weighted method(2).

Therefore, the inverse variance weighted (IVW) method was selected as the primary estimation approach (3). Being sensitive to several factors, including consistency, heterogeneity, and pleiotropy, there is a need for some supplementary approaches as sensitivity analysis(4)(5). To assess the robustness of the results, we applied four of these approaches (6), including MR-Egger, weighted median model, simple and weighted mode methods, which have been proven to work well for quality control (7).

One of the most frequently used methods to explore pleiotropy is MR-Egger(8)(9). Unlike the IVW method, which keeps the regression line's intercept firmly fixed at zero, MR-Egger allows this intercept to vary. This variation helps estimate the average SNP effect on the outcome when the SNP effect on the exposure is zero, thereby providing insights into directional horizontal pleiotropy(9). If the intercept value is exactly zero, it indicates that the MR-Egger estimate is identical to the IVW estimate(10).

Unlike the MR-Egger approach, the weighted median estimator has the advantage of improved precision in the estimates(11). By sorting ratio estimates in ascending order and assigning weights based on their precision, then taking the weighted median as the final estimate, this method calculates the estimates(12). Weighted median relies on the assumption that at least 50% of the total instrument weight comes from valid variants(13). It is more likely to reach an accurate causal estimate compared to the IVW method, as it remains consistent with the true causal effect even when up to 50% of the variants are invalid(14).

In a weighted mode-based method for two-sample Mendelian Randomization, researchers calculate Wald ratio estimates for each SNP and smooth their distribution(15). The simple mode of this smoothed distribution gives the causal effect estimate, while the weighted mode refines it by incorporating the precision of each SNP(12).

In order to determine if the findings that were obtained were consistent across the different studies and to assess the amount of heterogeneity, we applied Cochran's Q test for MR-inverse-variance weighted analyses to detect heterogeneity, Rucker's Q statistic for MR-Egger(16) and I^2 index(17). Cochran's Q statistic uses first-order weights to assess heterogeneity in MR. These weights reveal inconsistencies among instruments, indicating potential pleiotropic effects(18). Using the I^2 statistic enables us to assess the heterogeneity by knowing that values of <25%, 25–75%, and >75% represent low, moderate, and high levels of heterogeneity, respectively(19).

To detect and correct for horizontal pleiotropy through a regression-based framework, MR-PRESSO, a method in MR, was used (20,21). Utilising this method, the effects of genetic variants on the outcome are regressed on their effects on exposure to estimate the causal effect. It includes three main tests: a global test for overall pleiotropy, an outlier test to remove pleiotropic variants and a distortion test to evaluate changes in causal estimates after outlier removal(22). Radial MR updates the traditional summary MR regression model by adjusting causal estimates based on the ratio and weight of each SNP. Cook's distance is a measure used in regression analysis to identify influential points that may disproportionately affect the model's estimates. Larger Cook's distance values indicate greater influence, meaning that the specific data point has more leverage in shaping the regression outcomes. This diagnostic tool helps detect outliers or points that may distort the results, aiding in ensuring the robustness of the analysis(10). A Studentized residual represents the residual from a regression model, scaled by an estimate of its standard error. It reflects how well the model fits that specific point, with larger values signalling more outlying observations(10). After detecting and removing any of those outlier SNPs according to the aforementioned methods, the analysis was re-conducted.

All statistical analyses in this file have been performed in R software, version 4.0.3, along with several specific packages including "TwoSampleMR", "MendelianRandomization", "MR-PRESSO", "RadialMR", "MRMix", "mr. raps", "mrclust", and "GMRP". We also used the mrrobust package in STATA, version 17. We presented our results as odds ratios (OR) with their 95% confidence intervals, along with the p-value, which was $p < 0.05$.

Sensitivity Analysis

The sensitivity analysis confirmed the robustness of the inverse variance weighted (IVW) method through several supplementary approaches(23). We utilized the simple median and penalized weighted median methods, which supported the stability of the IVW results by downweighing potentially problematic instruments(24). The penalized IVW, robust IVW, and penalized robust IVW methods further validated the robustness of the IVW estimates by addressing potential biases and heterogeneity(25).

Additional analyses were conducted using penalized MR-Egger, robust MR-Egger, and penalized robust MR-Egger, which confirmed the absence of directional horizontal pleiotropy and addressed concerns of pleiotropy and heterogeneity(24). MR-RAPS, with L2, Huber, and Tukey loss

functions, ensured a minimal impact from outliers on the causal estimates (26) that can also account for weak instruments bias (27).

MR-Lasso and constrained maximum likelihood methods (MRcML) with configurations cML-MA-BIC-DP and cML-MA-BIC reinforced the robustness of the IVW results by enhancing instrument selection and model specification(28). The debiased inverse-variance weighted (dIVW)(29) method and mode-based method (MBE)(2) corroborated the causal effect estimates, while MRMix and contamination mixture method (ConMix)(30) analyses supported the findings by addressing mixture distributions among SNPs. Maximum-likelihood estimation further affirmed the robustness of the results. The true causative direction of the relationship was confirmed by the MR Steiger test ($P_{Steiger} < 0.05$)(31).

The results of the sensitivity analysis were further supported by a variety of visualization techniques, including forest, leave-one-out, funnel, and scatter plots(1). The Forest Plot displayed the individual causal estimates for each SNP, highlighting the overall effect and its consistency across instruments(32). The Leave-One-Out plot assessed the influence of individual SNPs by sequentially excluding one SNP at a time, confirming that no single SNP disproportionately affected the causal estimate(33). The Funnel Plot examined the symmetry of the effect estimates to detect potential directional pleiotropy, ensuring that any bias was minimal(1). A scatter plot provided a visual representation of the SNP-exposure and SNP-outcome associations, illustrating the linearity and strength of the instrumental variables used in the analysis(34). Together, these plots visually confirmed the robustness of the causal estimates and minimized concerns of pleiotropy, heterogeneity, or outlier bias. Lastly, we explored clustered heterogeneity to determine if groups of variants collaborate to affect traits (35).

1. Bowden J, Davey Smith G, Burgess S. Mendelian randomization with invalid instruments: effect estimation and bias detection through Egger regression. Int J Epidemiol. 2015;44(2):512–25.

2. Burgess S, Smith GD, Davies NM, Dudbridge F, Gill D, Glymour MM, et al. Guidelines for performing Mendelian randomization investigations: update for summer 2023. Wellcome open Res. 2019;4.

3. Huang G, Li W, Zhong Y, Liao W, Zhang Z. Mendelian randomization to evaluate the causal relationship between liver enzymes and the risk of six specific bone and joint-related diseases. Front Immunol. 2023;14:1195553.

4. Wan B, Ma N, Lu W. Evaluating the causal relationship between five modifiable factors and the

risk of spinal stenosis: a multivariable Mendelian randomization analysis. PeerJ. 2023;11:e15087.

5. Bowden J, Hemani G, Davey Smith G. Invited Commentary: Detecting Individual and Global Horizontal Pleiotropy in Mendelian Randomization-A Job for the Humble Heterogeneity Statistic? Am J Epidemiol. 2018 Dec;187(12):2681–5.

6. Fan J, Jiang T, He D. Genetic link between rheumatoid arthritis and autoimmune liver diseases: A two-sample Mendelian randomization study. Semin Arthritis Rheum. 2023 Feb;58:152142.

7. Sun M, Gao M, Luo M, Wang T, Zhong T, Qin J. Association between air pollution and primary liver cancer in European and east Asian populations: a Mendelian randomization study. Front public Heal. 2023;11:1212301.

8. Wang B, Zhang X, Liu D, Zhang J, Cao M, Tian X, et al. The Role of C-Reactive Protein and Fibrinogen in the Development of Intracerebral Hemorrhage: A Mendelian Randomization Study in European Population. Front Genet. 2021;12:608714.

9. Hartley AE, Power GM, Sanderson E, Smith GD. A guide for understanding and designing mendelian randomization studies in the musculoskeletal field. JBMR plus. 2022;6(10):e10675.

10. Burgess S, Thompson SG. Interpreting findings from Mendelian randomization using the MR-Egger method. Eur J Epidemiol. 2017;32(5):377–89.

11. Lee YH. Overview of Mendelian Randomization Analysis. J Rheum Dis. 2020;27(4):241–6.

12. Boehm FJ, Zhou X. Statistical methods for Mendelian randomization in genome-wide association studies: A review. Comput Struct Biotechnol J. 2022;20:2338–51.

13. Bowden J, Davey Smith G, Haycock PC, Burgess S. Consistent estimation in Mendelian randomization with some invalid instruments using a weighted median estimator. Genet Epidemiol. 2016;40(4):304–14.

14. Wootton RE, Lawn RB, Millard LAC, Davies NM, Taylor AE, Munafò MR, et al. Evaluation of the causal effects between subjective wellbeing and cardiometabolic health: mendelian randomisation study. BMJ. 2018 Sep;362:k3788.

15. Hartwig FP, Davey Smith G, Bowden J. Robust inference in summary data Mendelian randomization via the zero modal pleiotropy assumption. Int J Epidemiol. 2017 Dec;46(6):1985–98.

16. Bowden J, Del Greco M F, Minelli C, Zhao Q, Lawlor DA, Sheehan NA, et al. Improving the accuracy of two-sample summary-data Mendelian randomization: moving beyond the NOME assumption. Int J Epidemiol. 2019;48(3):728–42.

17. He Q, Bennett AN, Fan B, Han X, Liu J, Wu KCH, et al. Assessment of Bidirectional Relationships between Leisure Sedentary Behaviors and Neuropsychiatric Disorders: A Two-Sample Mendelian Randomization Study. Genes (Basel). 2022 May;13(6).

18. Hemani G, Bowden J, Davey Smith G. Evaluating the potential role of pleiotropy in Mendelian randomization studies. Hum Mol Genet. 2018;27(R2):R195–208.

19. Ojeda-Aravena A, Herrera-Valenzuela T, Valdés-Badilla P, Báez-San Martín E, Thapa RK, Ramirez-Campillo R. A Systematic Review with Meta-Analysis on the Effects of Plyometric-Jump Training on the Physical Fitness of Combat Sport Athletes. Sport (Basel, Switzerland). 2023 Jan;11(2).

20. Zhu X. Mendelian randomization and pleiotropy analysis. Quant Biol (Beijing, China). 2021 Jul;9(2):122–32.

21. Zhao J, Liang R, Song Q, Song S, Yue J, Wu C. Investigating association between gut microbiota and sarcopenia-related traits: a Mendelian randomization study. Precis Clin Med. 2023 Jun;6(2):pbad010.

22. Verbanck M, Chen C-Y, Neale B, Do R. Detection of widespread horizontal pleiotropy in causal relationships inferred from Mendelian randomization between complex traits and diseases. Nat Genet. 2018;50(5):693–8.

23. Shen C, Li X, Li L, Were MC. Sensitivity analysis for causal inference using inverse probability weighting. Biom J. 2011 Sep;53(5):822–37.

24. Rees JMB, Wood AM, Dudbridge F, Burgess S. Robust methods in Mendelian randomization via penalization of heterogeneous causal estimates. PLoS One. 2019;14(9):e0222362.

25. Burgess S, Zuber V, Gkatzionis A, Foley CN. Modal-based estimation via heterogeneity-penalized weighting: model averaging for consistent and efficient estimation in Mendelian randomization when a plurality of candidate instruments are valid. Int J Epidemiol. 2018 Aug;47(4):1242–54.

26. Mosteller F, Tukey JW. Data analysis and regression. A second course in statistics. Addison-Wesley Ser Behav Sci Quant methods. 1977;

27. Denault WRP, Bohlin J, Page CM, Burgess S, Jugessur A. Cross-fitted instrument: A blueprint for one-sample Mendelian randomization. PLoS Comput Biol. 2022 Aug;18(8):e1010268.

28. Tao Y, Wang Y, Yin Y, Zhang K, Gong Y, Ying H, et al. Associations of lipids and lipid-modifying drug target genes with atrial fibrillation risk based on genomic data. Lipids Health Dis. 2024 Jun;23(1):175.

29. Ye T, Shao J, Kang H. Debiased inverse-variance weighted estimator in two-sample summary-data mendelian randomization. Ann Stat. 2021;49(4):2079–100.

30. Burgess S, Foley CN, Allara E, Staley JR, Howson JMM. A robust and efficient method for Mendelian randomization with hundreds of genetic variants. Nat Commun. 2020;11(1):1–11.

31. Hemani G, Tilling K, Davey Smith G. Orienting the causal relationship between imprecisely measured traits using GWAS summary data. PLoS Genet. 2017;13(11):e1007081.

32. Chen Y, Zeng L. Peripheral Inflammatory Factors and Acute Myocardial Infarction Risk: A Mendelian Randomization Study. Glob Heart. 2023;18(1):55.

33. Dong H, Kong X, Wang X, Liu Q, Fang Y, Wang J. The Causal Effect of Dietary Composition on the Risk of Breast Cancer: A Mendelian Randomization Study. Nutrients. 2023 May;15(11).

34. Taschler B, Smith SM, Nichols TE. Causal inference on neuroimaging data with Mendelian randomisation. Neuroimage. 2022;258:119385.

35. Foley CN, Mason AM, Kirk PDW, Burgess S. MR-Clust: clustering of genetic variants in Mendelian randomization with similar causal estimates. Bioinformatics. 2021;37(4):531–41.
