# Supplementary material for: Genetic predisposition to serum 25 hydroxyvitamin D concentrations does not influence the risk of decreasing celiac disease in European ancestry: Evidence from meta-analysis and Mendelian randomization
Source: Medicine (Baltimore). 2026 Jul 3;105(27):e49587. doi: 10.1097/MD.0000000000049587 (PMC13336962; doi:10.1097/MD.0000000000049587)
Supplement: Supplementary file 2 [file medi-105-e49587-s002.pdf]

Figure S2. Forest plot of meta-analysis

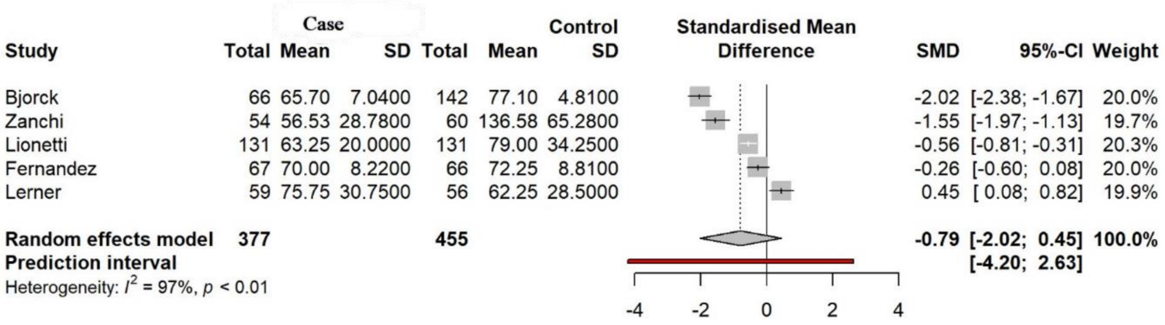

Forest plots are a key visualization technique used in meta-analyses to display the results of individual studies along with the overall effect estimate. Each study's effect size estimate is represented by a horizontal line, with the midpoint being the point estimate and the ends showing the confidence interval. The overall effect estimate is represented by a diamond shape at the bottom of the plot, indicating the combined effect across all studies. Confidence intervals, represented by horizontal lines extending from each study estimate, show the range of plausible values. The reference line at 0 indicates no effect. Here, details on the meta-analytical method, including the Inverse variance method (IVW), restricted maximum-likelihood estimator for tau2, Q-Profile method for confidence interval of tau2 and tau, Hartung-Knapp adjustment for the random effects model (df = 4), and Hedges' g (bias-corrected standardised mean difference; using exact formulae).

The plot indicates significant heterogeneity among the studies ($I^2 = 97\%$, $p < 0.01$). This means that there is substantial variability in the effect sizes across the studies. The SMD is -0.79. However, the wide 95% prediction interval (-4.20 to 2.63) indicates substantial uncertainty in this pooled estimate.
