# Supplementary material for: Genetic predisposition to serum 25 hydroxyvitamin D concentrations does not influence the risk of decreasing celiac disease in European ancestry: Evidence from meta-analysis and Mendelian randomization
Source: Medicine (Baltimore). 2026 Jul 3;105(27):e49587. doi: 10.1097/MD.0000000000049587 (PMC13336962; doi:10.1097/MD.0000000000049587)
Supplement: Supplementary file 3 [file medi-105-e49587-s003.pdf]

Figure S3. Leave-one-out plot of meta-analysis

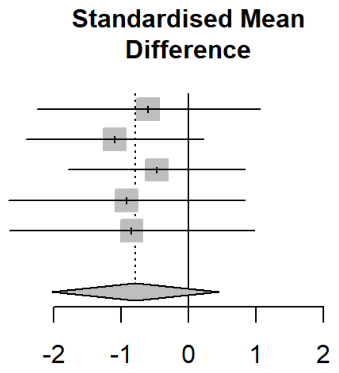

Study	SMD	95%-CI	P-value	Tau2	Tau	I2
Omitting Zanchi	-0.60	[-2.25; 1.05]	0.33	1.0456	1.0225	97%
Omitting Lerner	-1.09	[-2.41; 0.22]	0.08	0.6528	0.8079	96%
Omitting Bjorck	-0.48	[-1.79; 0.83]	0.33	0.6372	0.7982	94%
Omitting Fernandez	-0.92	[-2.67; 0.83]	0.19	1.1715	1.0823	97%
Omitting Lionetti	-0.84	[-2.66; 0.97]	0.24	1.2679	1.1260	97%
**Random effects model**	**-0.79**	**[-2.02; 0.45]**	**0.15**	**0.9547**	**0.9771**	**97%**

Leave-one-out analysis provides valuable diagnostics for assessing the robustness of meta-analysis results and identifying potentially influential studies. Details on meta-analytical method here, including inverse variance method, restricted maximum-likelihood estimator for tau^2, and Hartung-Knapp adjustment for random effects model (df = 3, 4).

The forest plot presents a leave-one-out analysis, examining the impact of excluding individual studies (Zanchi, Lerner, Bjorck, Fernandez, Lionetti) on the overall standardized mean difference (SMD) in a meta-analysis. The diamond shape represents the pooled estimate from the random effects model. The leave-one-out analysis shows that excluding any single study does not substantially alter the pooled estimate. The pooled estimate from the random effects model is -0.79, with a 95% CI of (-2.02, 0.45). This suggests a negative effect, but the wide CI indicates uncertainty in the precise magnitude of the effect.
