# Supplementary material for: Genetic predisposition to serum 25 hydroxyvitamin D concentrations does not influence the risk of decreasing celiac disease in European ancestry: Evidence from meta-analysis and Mendelian randomization
Source: Medicine (Baltimore). 2026 Jul 3;105(27):e49587. doi: 10.1097/MD.0000000000049587 (PMC13336962; doi:10.1097/MD.0000000000049587)
Supplement: Supplementary file 4 [file medi-105-e49587-s004.pdf]

Figure S4. Drapery plot

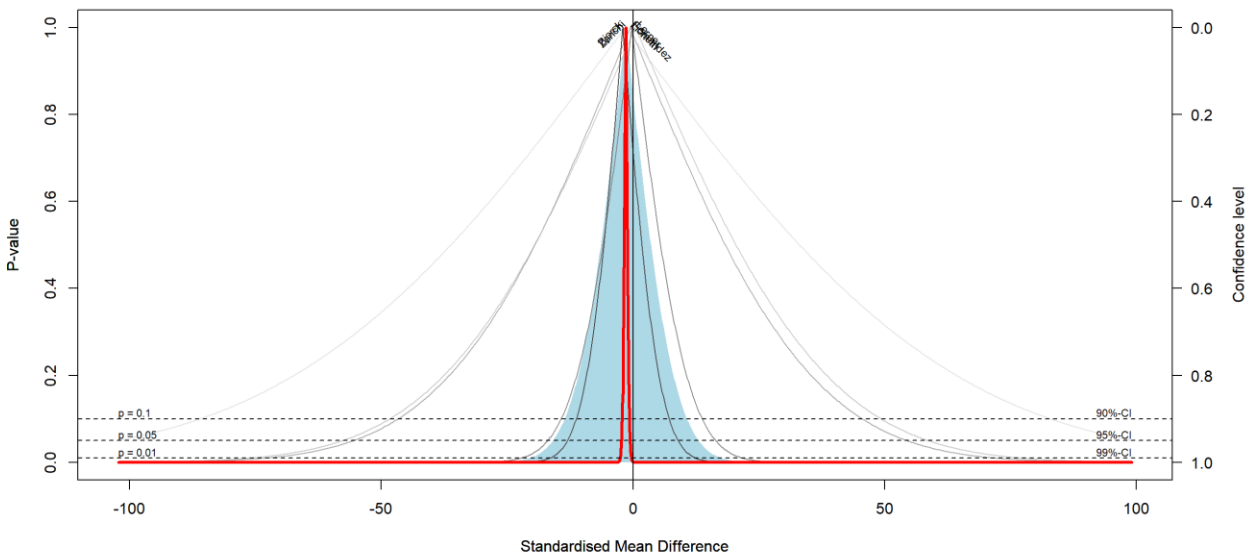

Forest plots are the most common way to visualize meta-analyses, and most researchers are familiar with their interpretation. It's recommended to include a forest plot in your meta-analysis report to provide a clear and concise summary of your findings. However, forest plots aren't the only visualization option. Meta-analyses can also be presented using drapery plots, which offer an alternative approach.

A limitation of forest plots is that they rely on a fixed significance threshold (typically $p < 0.05$) to determine statistical significance based on confidence intervals. Drapery plots utilize p-value functions to visualize the confidence interval for a range of p-values, rather than a single fixed threshold. This allows for a more continuous and flexible interpretation of the results. In a drapery plot, each study and the overall average effect are represented by a confidence curve. The x-axis shows the effect size, while the y-axis represents the p-value.
