# Supplementary material for: Genetic predisposition to serum 25 hydroxyvitamin D concentrations does not influence the risk of decreasing celiac disease in European ancestry: Evidence from meta-analysis and Mendelian randomization
Source: Medicine (Baltimore). 2026 Jul 3;105(27):e49587. doi: 10.1097/MD.0000000000049587 (PMC13336962; doi:10.1097/MD.0000000000049587)
Supplement: Supplementary file 5 [file medi-105-e49587-s005.pdf]

Figure S5. Funnel plot

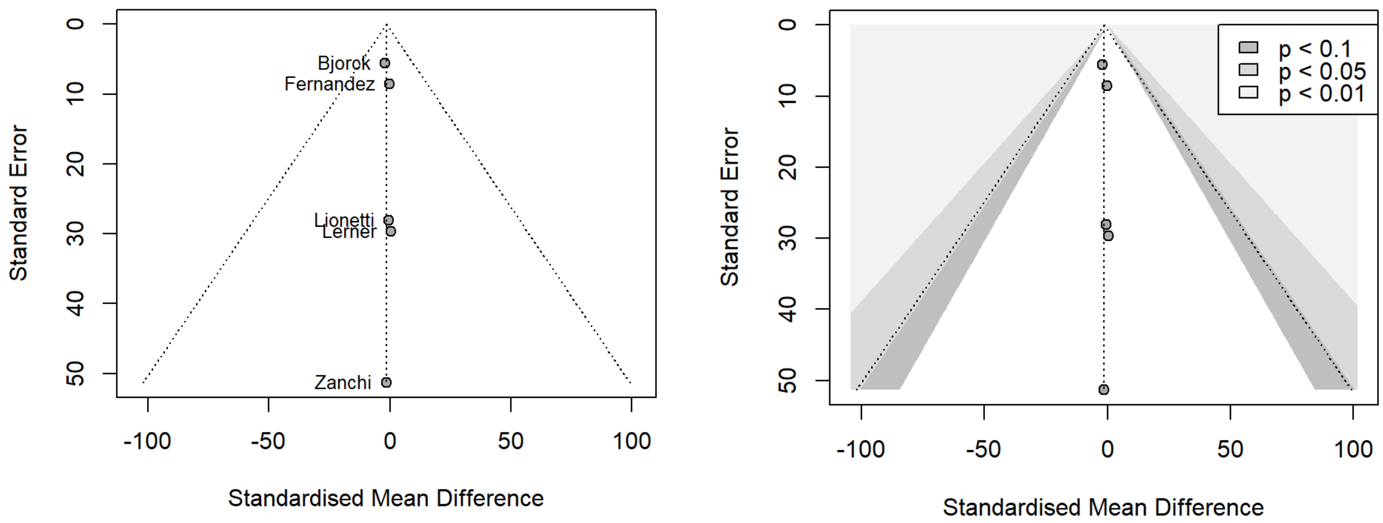

Based on the contour-enhanced funnel plot, there is a suggestion of potential publication bias. The funnel appears slightly asymmetrical, with a clustering of studies towards the left side. This suggests that studies with smaller or non-significant effect sizes might be underrepresented. As a result, the trim and fill method was employed to adjust the probability bias (Random effect model= -2.0264; 95%CI: -9.7419, 5.6891; Z=-0.51; P-value=6067). This result revealed no potential bias.
