# Supplementary material for: Genetic predisposition to serum 25 hydroxyvitamin D concentrations does not influence the risk of decreasing celiac disease in European ancestry: Evidence from meta-analysis and Mendelian randomization
Source: Medicine (Baltimore). 2026 Jul 3;105(27):e49587. doi: 10.1097/MD.0000000000049587 (PMC13336962; doi:10.1097/MD.0000000000049587)

Figure S7. Scatter plot showing the genotype summary level data points and the fitted MR-Egger model

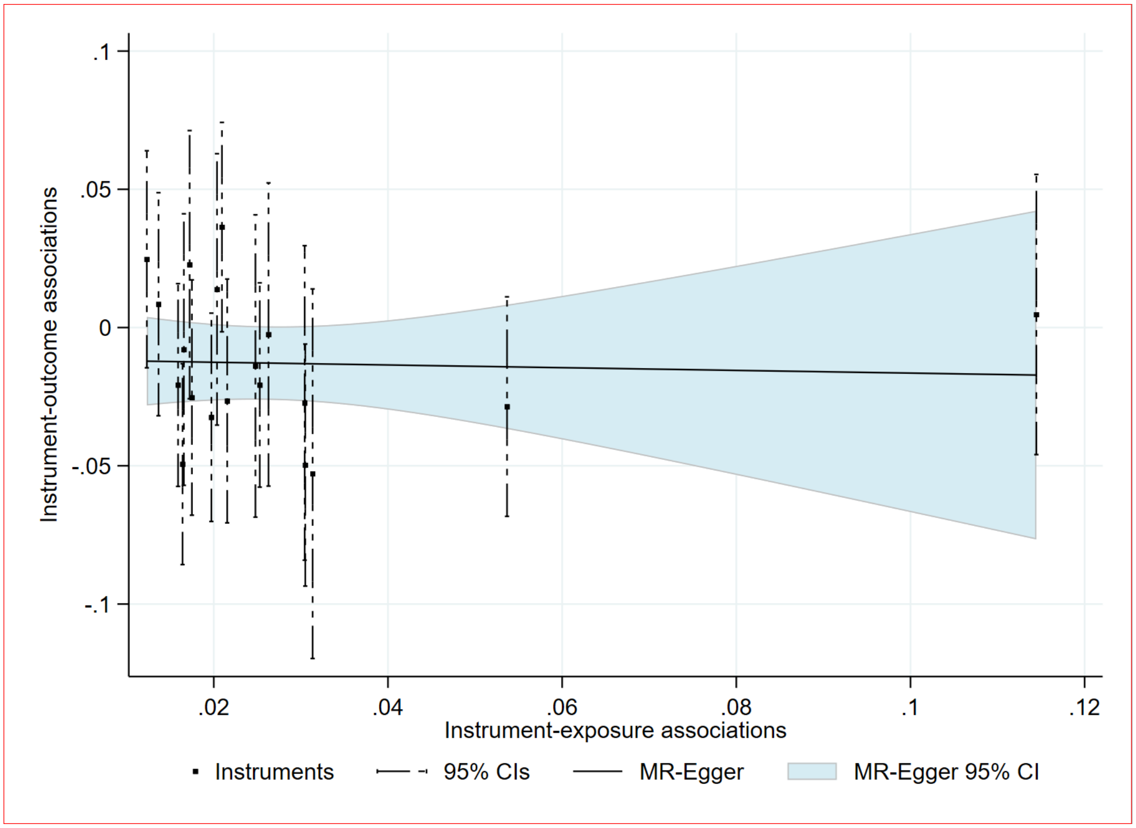

Supplement: Supplementary file 7 [file medi-105-e49587-s007.pdf]
