# Supplementary figures and images for: Genetic predisposition to serum 25 hydroxyvitamin D concentrations does not influence the risk of decreasing celiac disease in European ancestry: Evidence from meta-analysis and Mendelian randomization
Source: Medicine (Baltimore). 2026 Jul 3;105(27):e49587. doi: 10.1097/MD.0000000000049587 (PMC13336962; doi:10.1097/MD.0000000000049587)

**Figure S8. Leave-one-out plot**

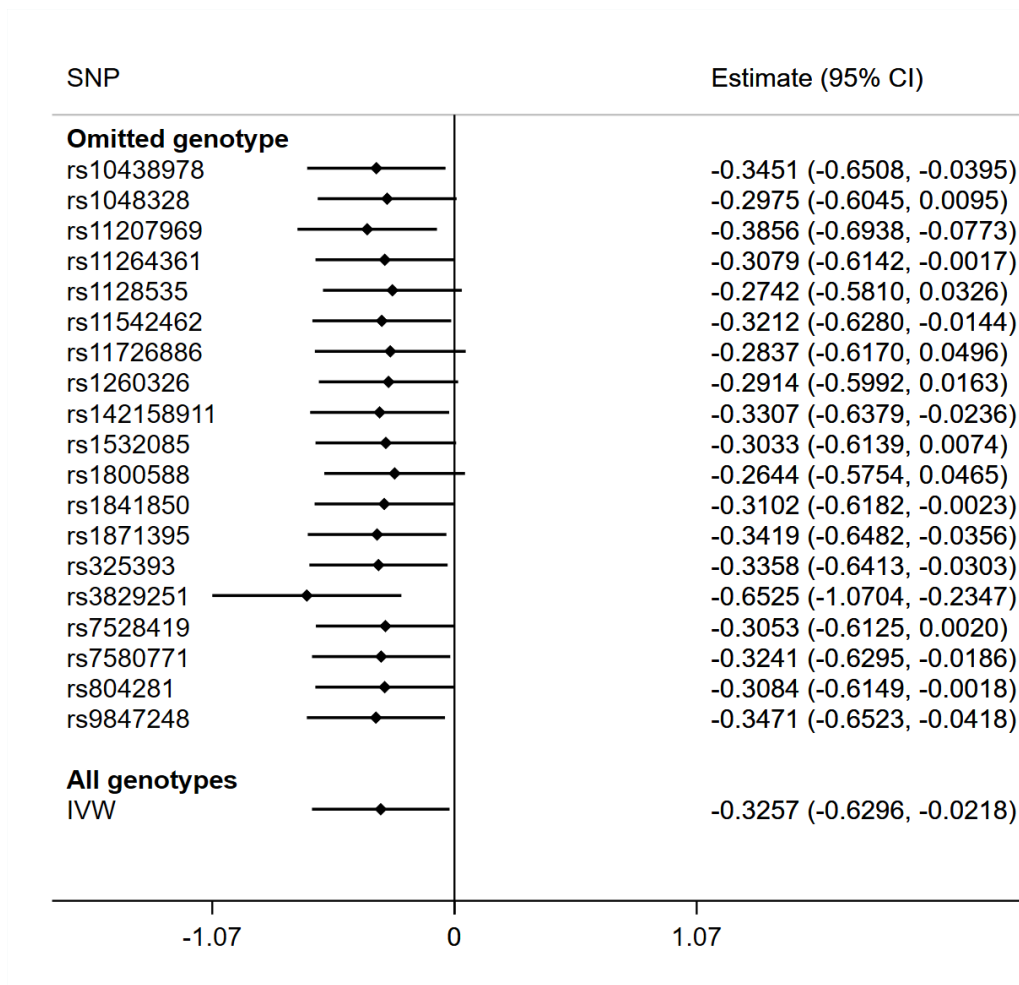

Supplement: Supplementary file 8 [file medi-105-e49587-s008.pdf]

Figure S9. Funnel plot

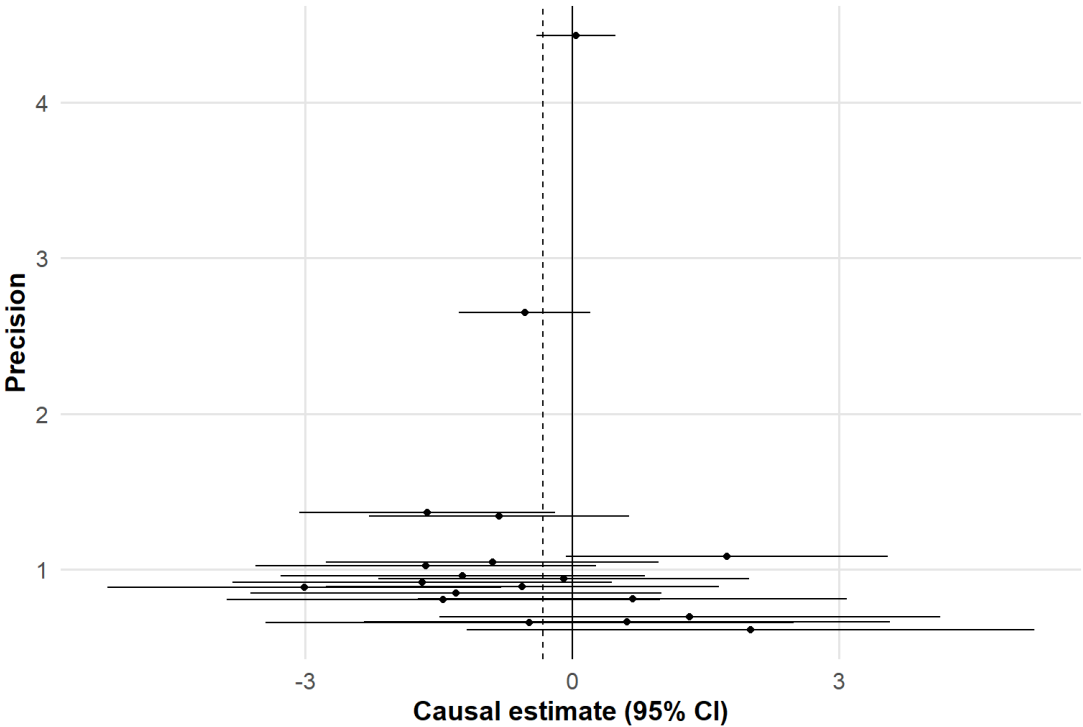

Supplement: Supplementary file 9 [file medi-105-e49587-s009.pdf]

**Figure S10. Forest plot**

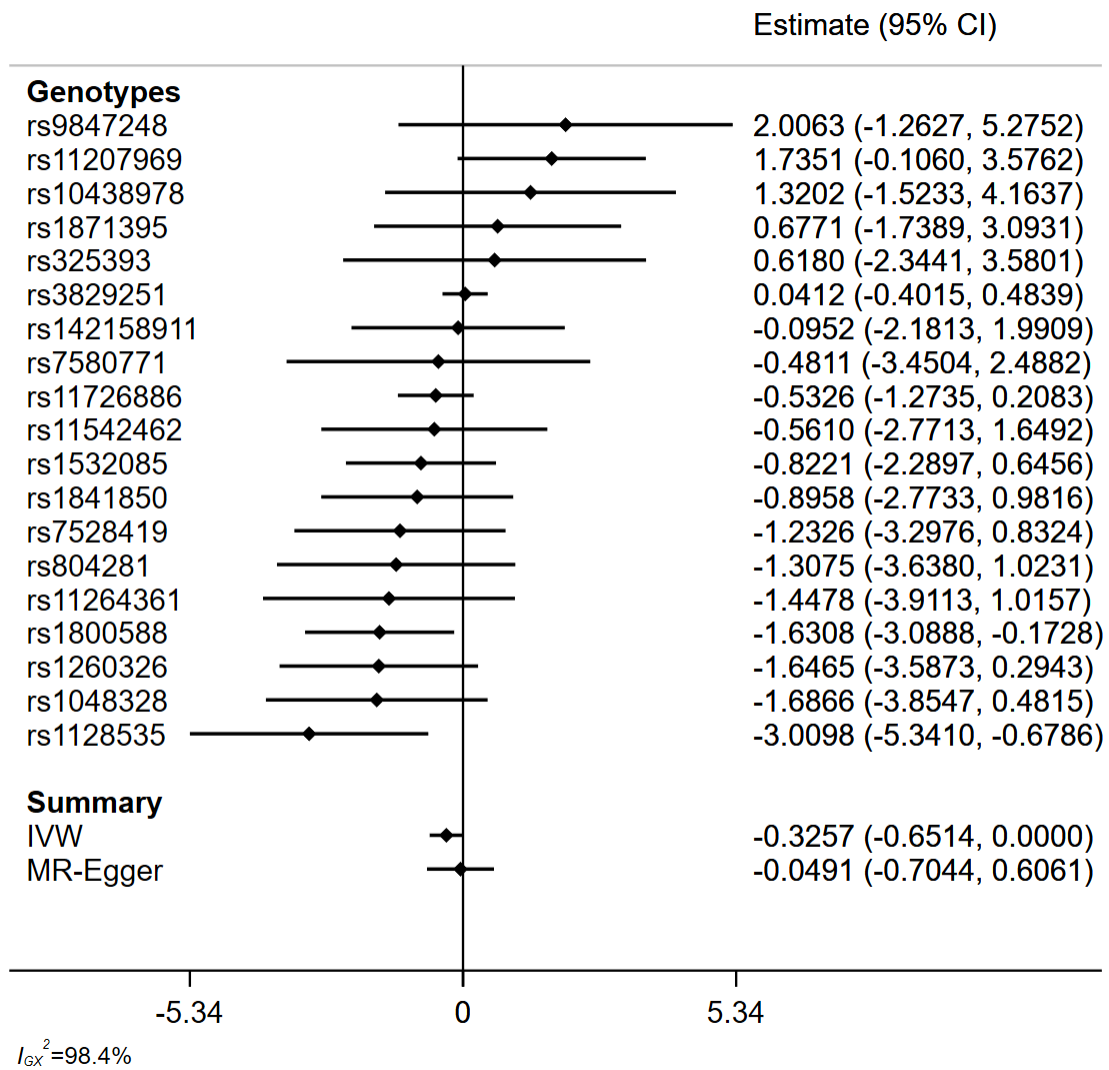

Supplement: Supplementary file 10 [file medi-105-e49587-s010.pdf]

**Figure S12. Steps of two-sample Mendelian randomization**

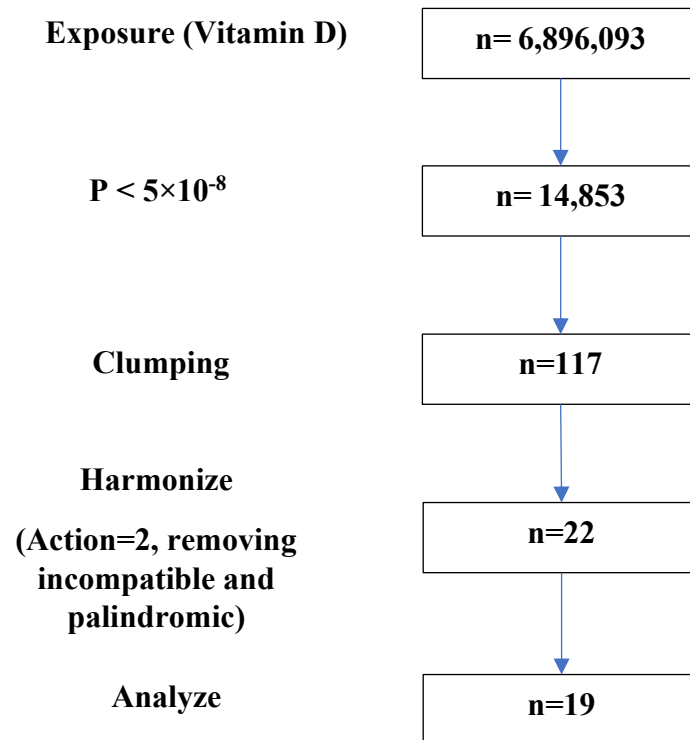

Supplement: Supplementary file 12 [file medi-105-e49587-s012.pdf]

**Figure S13. Steps of two-sample Mendelian randomization**

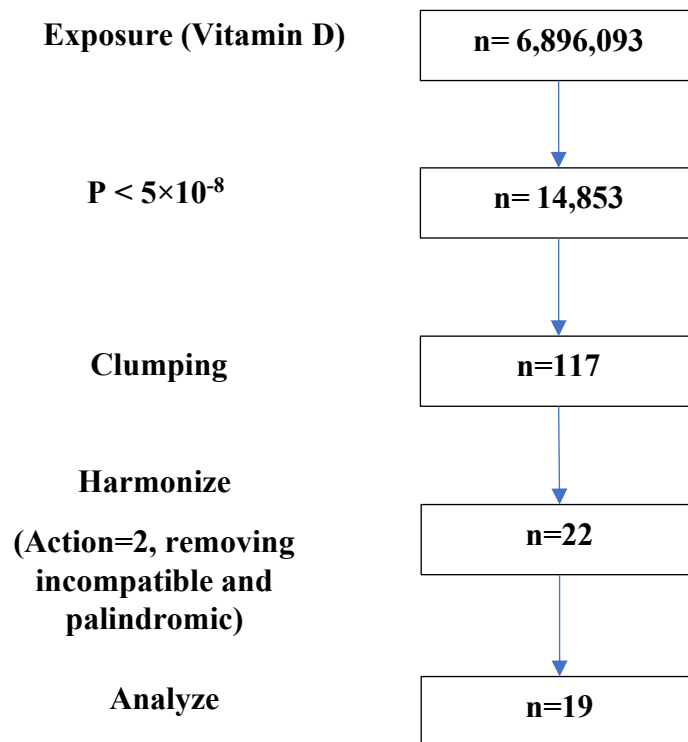

Supplement: Supplementary file 13 [file medi-105-e49587-s013.pdf]
