# Supplementary material for: Genetic predisposition to serum 25 hydroxyvitamin D concentrations does not influence the risk of decreasing celiac disease in European ancestry: Evidence from meta-analysis and Mendelian randomization
Source: Medicine (Baltimore). 2026 Jul 3;105(27):e49587. doi: 10.1097/MD.0000000000049587 (PMC13336962; doi:10.1097/MD.0000000000049587)
Supplement: Supplementary file 11 [file medi-105-e49587-s011.pdf]

Figure S11. Densities of the IV estimates using different values of the phi parameter

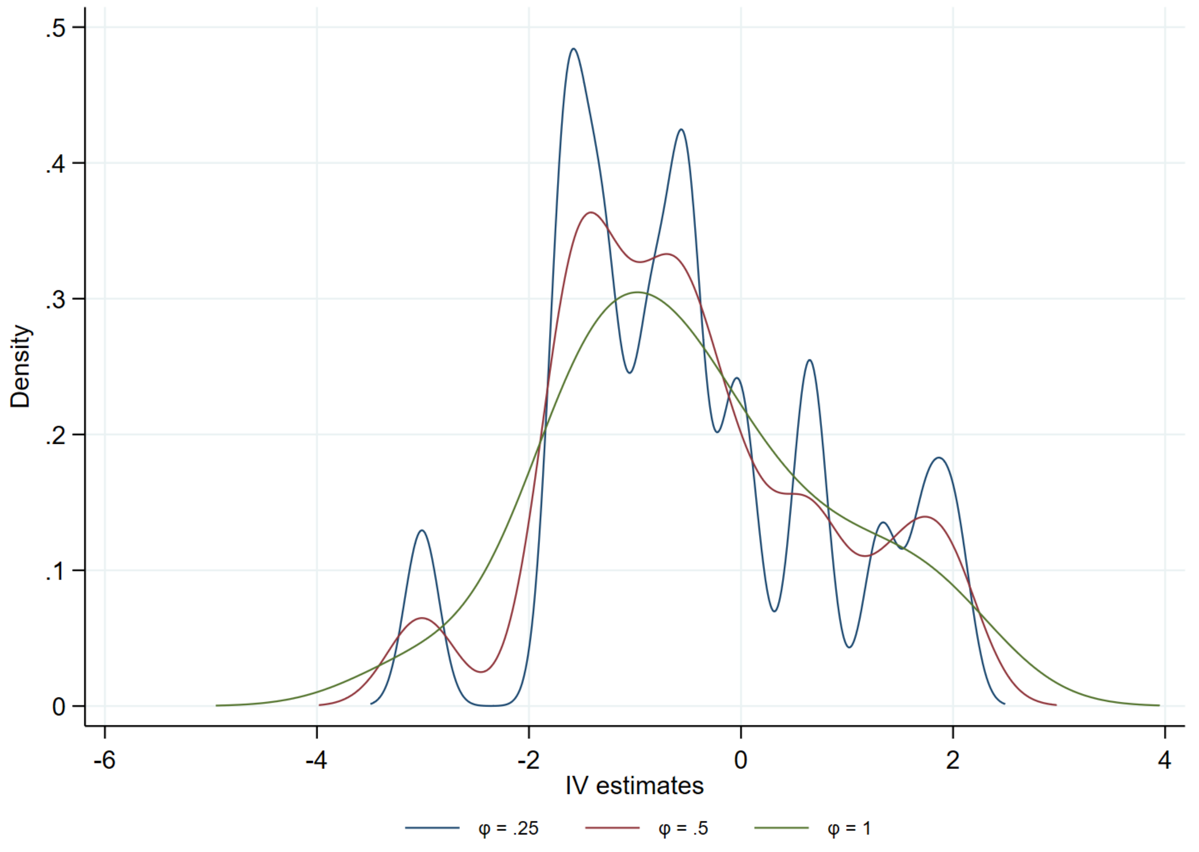

Estimates using different values of the phi parameter.

Phi	Beta	Standard Error	Z Statistic	P-value	95%CI
0.25	-1.574	0.799	-1.97	0.049	-3.141, -0.006
0.50	-1.421	0.714	-1.99	0.047	-2.822, -0.0202
1.00	-0.981	0.513	-1.91	0.056	-1.987, 0.025

The parameter φ controls the precision of the IV estimates. Higher values of φ result in more precise. The plot illustrates how the density of IV estimates changes with different values of φ, highlighting the trade-off between precision and variability in the estimates.
