# Supplementary material for: Genetic predisposition to serum 25 hydroxyvitamin D concentrations does not influence the risk of decreasing celiac disease in European ancestry: Evidence from meta-analysis and Mendelian randomization
Source: Medicine (Baltimore). 2026 Jul 3;105(27):e49587. doi: 10.1097/MD.0000000000049587 (PMC13336962; doi:10.1097/MD.0000000000049587)
Supplement: Supplementary file 14 [file medi-105-e49587-s014.pdf]

Figure S6. Display the relationship of the undefined causal variables between serum 25-hydroxyvitamin D concentration and celiac disease

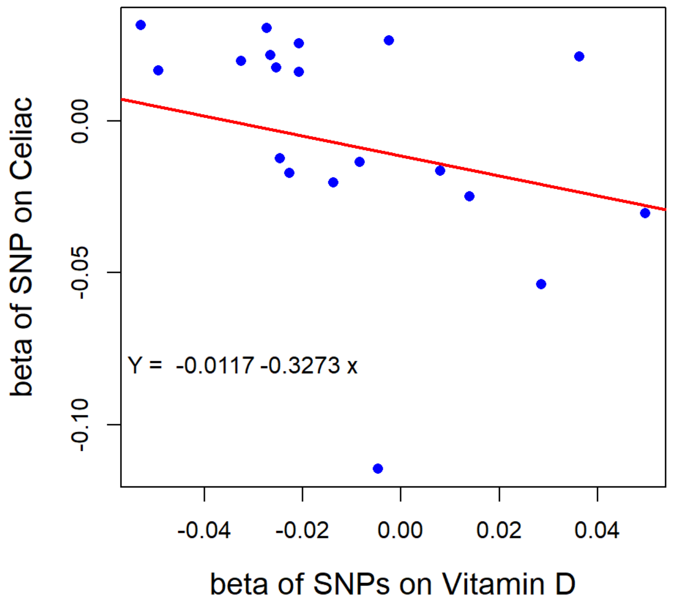

Coefficients	Estimate	Standard Error	T- value	P-value
Intercept	-0.011651	0.005862	-1.987	0.0632
Slop	-0.327260	0.164238	-1.993	0.0626
